# Host-parasitoid associations in marine planktonic time series: Can metabarcoding help reveal them?

**DOI:** 10.1371/journal.pone.0244817

**Published:** 2021-01-07

**Authors:** Laura Käse, Katja Metfies, Stefan Neuhaus, Maarten Boersma, Karen Helen Wiltshire, Alexandra Claudia Kraberg

**Affiliations:** 1 Alfred-Wegener-Institut, Helmholtz-Zentrum für Polar- und Meeresforschung, Biologische Anstalt Helgoland, Helgoland, Schleswig-Holstein, Germany; 2 Alfred-Wegener-Institut, Helmholtz-Zentrum für Polar- und Meeresforschung, Bremerhaven, Bremen, Germany; 3 Helmholtz-Institut für Funktionelle Marine Biodiversität, Oldenburg, Germany; 4 University of Bremen, Bremen, Bremen, Germany; 5 Alfred-Wegener-Institut, Helmholtz-Zentrum für Polar- und Meeresforschung, Wadden Sea Station, List auf Sylt, Schleswig-Holstein, Germany; IRIG-CEA Grenoble, FRANCE

## Abstract

In this study, we created a dataset of a continuous three-year 18S metabarcoding survey to identify eukaryotic parasitoids, and potential connections to hosts at the Long-Term Ecological Research station Helgoland Roads. The importance of parasites and parasitoids for food web dynamics has previously been recognized mostly in terrestrial and freshwater systems, while marine planktonic parasitoids have been understudied in comparison to those. Therefore, the occurrence and role of parasites and parasitoids remains mostly unconsidered in the marine environment. We observed high abundances and diversity of parasitoid operational taxonomic units in our dataset all year round. While some parasitoid groups were present throughout the year and merely fluctuated in abundances, we also detected a succession of parasitoid groups with peaks of individual species only during certain seasons. Using co-occurrence and patterns of seasonal occurrence, we were able to identify known host-parasitoid dynamics, however identification of new potential host-parasitoid interactions was not possible due to their high dynamics and variability in the dataset.

## Introduction

Parasitism is a common lifestyle for a wide variety of species, including planktonic ones. It is one of the multiple biotic factors that can influence food web structure. For example, there can be changes in food chain length, connectivity, and stability [1–3]. Such effects have previously been shown for planktonic freshwater systems [4, 5] but little information is available for the marine realm especially with regards to eukaryotic parasitoids [6, 7]. Parasitoids, those organisms that ultimately kill their hosts, in the marine environment range from viruses and bacteria to several protist taxa. Whereas some progress has been made in recent years on bacterial and viral infections [8–14], studies on eukaryotic parasites and parasitoids have focused mainly on single host-parasitoid/parasite systems (in the following only named as host-parasitoid systems) or species groups, in short-term microscopy-based projects [15–19]. Currently, long-term (multi-year) investigations are largely missing. These kind of investigations could yield important information on the dynamics of the interactions.

While it is known that infection by a parasite affects the fitness of the host and most parasites are transferred through several different hosts, parasitoids often complete their life cycle in a single host and kill the host in the process [20, 21]. Since protist parasites are often classified as parasitoids [22–24] and a definite distinction between parasites and parasitoids is difficult for some planktonic taxa, we will only use the term parasitoid in the following manuscript to describe all taxa that have been found to be related to the parasitism strategy. Parasitoid microbes can be drivers of phytoplankton bloom dynamics, play important roles in host population regulation [20, 21] and can influence phytoplankton succession due to their selectiveness of host species [25]. The infection by parasitoids can even cause a phytoplankton bloom to collapse [26–28]. For example, Tillmann et al. [25] indicated that parasitic infections of phytoplankton compete with zooplankton in marine food webs, as algal cells are killed and consequently no longer available to higher trophic levels such as mesozooplankton. Indeed, even classic Lotka-Volterra dynamics, defined as periodic and alternating fluctuations of predator and prey, have been observed in host-parasitoid relationships [29], and peaks in abundance of a host are followed by peaks in abundance of a parasitoid [30].

Even though the examples cited above may suggest otherwise, our knowledge on the role of parasitoids in marine ecosystems is still incomplete [20]. This paucity of information is strongly related to insufficient monitoring capacity and methodological constraints [20], and even the identification of organisms as parasitoids and their subsequent taxonomic determination is difficult and needs improvement. Otherwise, it is not possible to make some inference about the impact of parasitoids on marine ecosystems.

Considerable diversity exists in marine parasitoid protists and an equally diverse range of known hosts, including marine algae, nematodes, crustaceans and fish has been described [20]. So far, several eukaryotic taxa are known to include parasitoid classes: Dinoflagellata, Stramenopiles, Cercozoa, Ciliophora, Apicomplexa, Mesomycetozoa, Metazoa, Lobosa, Perkinsida and true Fungi. The hosts of many of those parasitoid protists are protists themselves. Syndiniales, for example, a class of dinoflagellates, is composed exclusively of parasitoid species, and occur globally, including the Arctic and Antarctic [31] and may, as a result, be rather abundant in metabarcoding datasets [32–34]. They can infect several hosts, ranging from dinoflagellates and ciliates to copepods, crabs and fish. For example they have been found to be lethal to the eggs or newly hatched fish larvae [35]. Another example of a class of mostly protistan parasitoids are the heterokont oomycetes. These belong to the kingdom of Stramenopiles [36, 37], and infect a wide range of hosts such as brown algae, diatoms, crustaceans and fish in marine environments [21]. While some parasitoids are host-specific, others can infect different (plurivorous) species, and in return, hosts can be infected by several parasitoids simultaneously [37].

As one of the longest running long-term observatories, Helgoland Roads Long-Term Ecological Research site (LTER) provides abiotic and biotic data at a very high temporal resolution, including phytoplankton, temperature, salinity and inorganic nutrients [38, 39]. During the course of this long-term observation programme at Helgoland, several diatom-infecting parasitoids were already detected, using light microscopic observation. These include Cryomonadida such as the nanoflagellate *Cryothecomonas aestivalis*, which is known to infect the diatom *Guinardia delicatula* [19, 27], the Oomycete *Lagenisma coscinodisci*, which is known to infect the diatom *Coscinodiscus* sp. [40, 41] and also two recently described oomycete parasitoid species: *Miracula helgolandica* in the host *Pseudo-nitzschia pungens* [24, 42] and *Olpidiopsis drebesii* in *Rhizosolenia imbricata* [42]. *Cryothecomonas longipes*, which can infect a broad spectrum of diatoms including *Thalassiosira rotula* [18], and several *Pirsonia sp*. with possible hosts like *Rhizosolenia sp*. [15] were detected in the North Sea but not yet at Helgoland.

As indicated above, most of the evidence on host-parasitoid interactions at Helgoland was derived from microscopic methods. However, many of the organisms involved are small and without conspicuous characteristics. They can -if at all- only be identified as flagellates in the pico- and nanoplankton fractions in their free living states or by spotting inside of infected host cells [37]. Therefore, there is great scope for improvement. Next generation sequencing (NGS) and other molecular methods have great potential to close this gap, but we do not know enough yet, to be able to implement these techniques in a long-terms series approach. Open questions are, for example, whether relevant temporal dynamics in a host-parasitoid system can be observed if the parasitoid changes from free living to parasitic stages. Furthermore, it also remains to be seen whether host-parasitoid dynamic behaviour follows the Lotka-Volterra type dynamics in a complex ecological context, with predators and competitors also present. The fact that several host-parasitoid systems have already been identified for Helgoland offers us the unique opportunity to test these open questions. It allows us to investigate the potential benefits and drawbacks of molecular methods in this context.

It was the aim of this study to create a high resolution and unique 18S metabarcoding dataset of continuous, high frequency sampling of three years duration (1) to identify the extent of planktonic eukaryotic parasitoid occurrence within the community at Helgoland Roads throughout the year, and potential links to environmental conditions. Furthermore, we want (2) to assess if it is possible to detect known host-parasitoid systems, which have been described by conventional microscope analysis, and their dynamics using the sequencing dataset. By using the knowledge gleaned from the dynamics analysis of (2), we aim to (3) examine if potential host-parasitoid systems, that are not known at Helgoland but elsewhere, can be detected with these data based upon identification of alternating cyclical dynamics, plus if dynamical behaviour of host-parasitoid pairs allows for the identification of thus far unknown host-parasitoid associations.

## Materials and methods

### Study site and sampling

We took water surface samples from the Helgoland Roads LTER sampling site. The sampling site (54°11.03’ N, 7°54.00’E) is situated between the main island of Helgoland and the dune island [38]. Secchi depth and temperature were measured directly. Other parameters include salinity, nutrients such as silicate, phosphate, inorganic nitrogen and chlorophyll, which were measured in the laboratory according to the LTER sampling protocol [38, 43, 44], for nutrients [45]. Daily observations of sunshine duration in hours were downloaded from the Deutscher Wetterdienst, Climate Data Centre [46]. Seasons were defined as follows: Spring = March to May, Summer = June to August, Autumn = September to November, Winter = December to February.

In total, three different sampling phases from the same station were combined to build a comprehensive dataset of over 3 years. In short, the first sampling phase was conducted from March 2016 to May 2016 (work-daily sampling) [47]. The second phase included samples from June to October 2016 (in total 6 samples, irregular sampling) [48]. The third phase was conducted from December 2016 until March 2019, where samples were taken twice a week. In the period between May to July 2018 we intensified sampling by increasing the frequency to three samples per week (see S1 Table for further information on the samples belonging to each sampling phase).

For sequencing, we filtered 1 L of the water sample. For the sampling phase 1, a sequential filtration was used as part of another sampling program for bacterial long-term monitoring [49, 50]. The sample was filtered through 10 μm polycarbonate filters, 3 μm PC filters and 0.2 μM polyvinylidene fluoride filters (Millipore, Schwalbach, Germany) according to the protocol by Teeling et al. [49]. Samples from sampling phase 2 and 3were filtered with 0.45 μm nylon filters (Whatman, 47 mm). Following filtration, all filters were immediately frozen at -20°C. It needs to be mentioned that the different pore sizes of the sampling phases do not influence the detection of the eukaryotic picoplankton, due to their general size being bigger than 0.45 μm.

### DNA-extraction

We used the Macherey-Nagel NucleoSpin® Plant II Kit for DNA extraction from the 10 μm, 3 μm of sampling phase 1 and all 0.45 μm filters from sampling phase 2 and 3, before the extracts were stored at -20°C. DNA extraction from 0.2 μm filters from sampling phase 1 was conducted as described previously by Sapp et al. [51]. In short, cells were lysed with lysozyme and sodium dodecyl sulfate, a phenol/chloroform/isoamyl alcohol solution was used for DNA extraction with isopropanol used in the precipitation step. Here the DNA was eluted in sterile water. Then we pooled the separate DNA extracts from the sequentially filtered samples to obtain one sample per sampling date. The nucleic acid content of all samples was measured with a Quantus Fluorometer using the QuantiFluor® dsDNA System (Promega, USA).

### MiSeq™ Illumina sequencing and data processing

We used the Nextera XT DNA Library Preparation protocol (Illumina, USA) to prepare the DNA isolates for the MiSeq™ Illumina sequencing. We identified a fragment of the V4 region of the 18S rDNA using the following primer set: 528iF (GCG GTA ATT CCA GCT CCA A) and 964iR (AC TTT CGT TCT TGA TYR R) [52]. For polymerase chain reactions (PCRs) KAPA HiFi HotStartReadyMix (Kapa Biosystems, Inc., USA) was used to avoid contamination. Afterwards, we confirmed the success of this amplicon PCR by using 2 μL of the PCR product for gel electrophoresis. 5 additional cycles were added to the original PCR program, if an increase of template (up to 5 μL) was not sufficient. About 43 million 2x300 bp paired-end sequences were produced using an Illumina MiSeq™ sequencer (Illumina, USA).

We then used our in-house developed pipeline for bioinformatic processing of the samples as described below (for more information see S1 File and https://github.com/PyoneerO/qzip).

The low-quality 3'-ends of the reads were trimmed by *Trimmomatic* (version 0.38) [53] and the paired-ends were merged by *VSEARCH* (version 2.3.0) [54]. *Cutadapt* (version 1.19) [55] was used to adjust the sequence orientation and to remove the forward and reverse primer matching sequence segments. Sequences were only kept if both primer matching segments could be detected. The remaining sequences were filtered by *VSEARCH* and sequences were discarded, i) if they were shorter than 300 bp or longer than 550 bp, ii) if they carried any ambiguity or iii) if the expected base error (sum of all base error probabilities) of a sequence was above 0.25.

Chimeric sequences were sample-wise predicted by *VSEARCH* in *de novo* mode with default settings and removed from the sample files. Only samples with at least 10000 sequences after filtering were considered for further analyses.

The remaining 21 million sequences were clustered into operational taxonomic units (OTUs) by the tool s*warm* (version 2.2.2) [56, 57] with default settings. For each OTU the most abundant amplicon was selected as representative and taxonomically annotated with the default classifier implemented in *mothur* (version 1.38.1) [58]. As reference the *Protist Ribosomal Reference database* (PR2), version 4.11.1 [59], was chosen and the minimum confidence cut-off for annotation was set to a value of 80. The sequence data is available in the European Nucleotide Archive (ENA) at the European Bioinformatics Institute (EMBL-EBI) under accession number PRJEB37135 (https://www.ebi.ac.uk/ena/data/view/PRJEB37135), using the data brokerage service of the German Federation for Biological Data (GFBio) [60], in compliance with the Minimal Information about any (X) Sequence (MIxS) standard [61].

### Data analysis and statistics

We reviewed the entire dataset of all 59,284 OTUs (in total 20,476,979 reads) for parasitoid taxa. For this we used information of literature focusing on known parasitoids in the North Sea and of the Tara Oceans Database W3 from the Companion Website of the article of de Vargas et al. [22]. Afterwards a threshold of 0.001% of total reads was applied to the full dataset. Hereby all OTUs remained, which had a total read count of 205 or higher, resulting in a limited dataset of 2790 OTUs. Out of this dataset, parasitoids that are known to be parasitizing plankton were extracted to get an overview of present parasitoids. Host-parasitoid relationships were identified by comparing occurrences of several parasitoids with potential hosts as described in the literature. Here, we defined peaks as local maxima during a certain period. The relative abundance needed to be at least 10% or more of the maximum relative abundance of the respective OTU or group. For diatom hosts, the word bloom was used, if various peaks could be identified in several consecutive samples or if high abundances above 10% were reached. Our goal was to find the relationships in the first place rather than describing the dynamics as a model. Also distinct time lags between host and parasitoid occurrence are either unknown for known relationships or can not be assumed to be correct for new potential relationships. Therefore, we focused on identifying two cases: 1. Alternating associations of potential hosts and parasitoid were considered to indicate typical Lotka-Volterra dynamics of the host-parasitoid system and time lags of up to several days as they have been identified by microscopic analysis in the past. 2. Simultaneous appearance of potential host and parasitoid were expected to indicate a current infection.

For investigation of new host-parasitoid relationships two different approaches were tested. Parasitoid occurrences were compared with different hosts as they are known from the literature from other areas as well as closely related species. The limited dataset (2790 OTUs) was used to identify potential relationships that were found to be relevant based on the two cases of identification as described above. By using the known sequences, parasitoid OTUs and their possible hosts were verified with the Basic Local Alignment Search Tool (BLAST), when specific host-parasitoid systems were investigated.

A constrained ordination model based on the OTU table (based on relative abundances) and available environmental parameters was conducted in R, version 4.0.0 [62], using the vegan package [63]. Seasons and total parasitoid occurrence (as relative abundance) were included as additional parameters. Single parameters were combined with an analysis of variance-like permutation test for Canonical Correspondence Analysis (CCA) to assess the significance of the constraining factors [63]. The variables were chosen by their significance (p <0.05). If several variables were given as significant in the same step, the variable with the lowest Akaike information criterion (AIC) value was chosen to minimize the information loss [64]. Environmental parameters that were included in the model development were temperature, salinity, Secchi depth, tide and sunshine duration as well as silicate, phosphate and nitrate concentrations. Due to missing parameters on seven different sampling dates (phosphate: 4 dates; silicate, nitrate temperature and salinity: 1 date each) the analysis was conducted with 273 samples.

## Results

### Baseline survey of parasitoid diversity

The 280 samples of the entire 18S metabarcoding dataset included 59,284 OTUs in total, of which 6056 OTUs (10.2%) were identified as potential parasitoids based on literature (see S2 Table for sequencing statistic). Over 55 percent of the dataset remained of unknown trophic mode due to insufficient taxonomic identification or missing reports on trophic modes. After setting a threshold of 0.001% of total reads, 2790 OTUs remained, of which 461 (16.5%) were identified as potential parasitoids based on their taxonomy and literature knowledge (S3 Table, see S1 File for comparison of results of different pipeline settings). For at least 124 parasitoid OTUs occurrence of taxa were known for Helgoland or nearby regions in the North Sea. Additionally, the assignment of parasitism or other trophic modes was not possible for at least 50 percent of the remaining OTUs, which shows that there is still a great need for autecological studies on the plankton. Total reads of parasitoids were about ten times lower than non parasitoid reads and total relative abundances of parasitoids reached up to 45% (S1 Fig).

### Parasitoid diversity, succession and influence of environmental conditions

The parasitoid OTUs belonged to ten different phyla (Table 1). These could be divided into 15 different classes, which are known to infect a wide range of hosts.

**Table 1:** Overview of parasitoid diversity on phylum and class level.

Phylum	OTU Count	Classes	Known hosts	References
Dinoflagellata	206	Syndiniales	Radiolaria, Dinoflagellata, Ciliates, Crustacea like Copepoda and Amphipoda, Cnidaria, Fish eggs, Chaetognatha	[20, 26, 31, 32, 65, 66]
Cercozoa	140	Endomyxa, Endomyxa-Phytomyxea, Filosa-Imbricatea, Filosa-Thecofilosea	Green plants, Brown algae, Diatoms and Stramenopiles	[18, 19, 27, 37, 67–72]
Stramenopiles_X	51	Oomycota, Pirsonia_Clade	Diatoms, Crustacea, Macro algae, Fish	[15, 17, 20, 23, 25, 36, 41, 73, 74]
Fungi	20	Ascomycota, Chytridiomycota	Cyanobacteria, Diatoms	[21, 75]
Apicomplexa	19	Apicomplexa_X	Arthropoda, Polychaeta, Chaetognatha, Copepoda, Euphausiacea, Dinoflagellata	[20, 31, 76]
Mesomycetozoa	14	Ichthyosporea	Diatoms, Fish, Mollusca Crustaceae	[77–79]
Ciliophora	5	Oligohymenophorea	Copepoda, Euphausiacea, Chaetognatha,	[80–83]
Metazoa	3	Nematoda	Hexapoda, Mollusca, Clitellata, Myriapoda, Crustacea, Annelida, Arthropoda	[84]
Lobosa	2	Tubulinea	Diatoms	[77]
Perkinsea	1	Perkinsida	Mollusca, Dinoflagellata	[85, 86]

**Fig 1:**
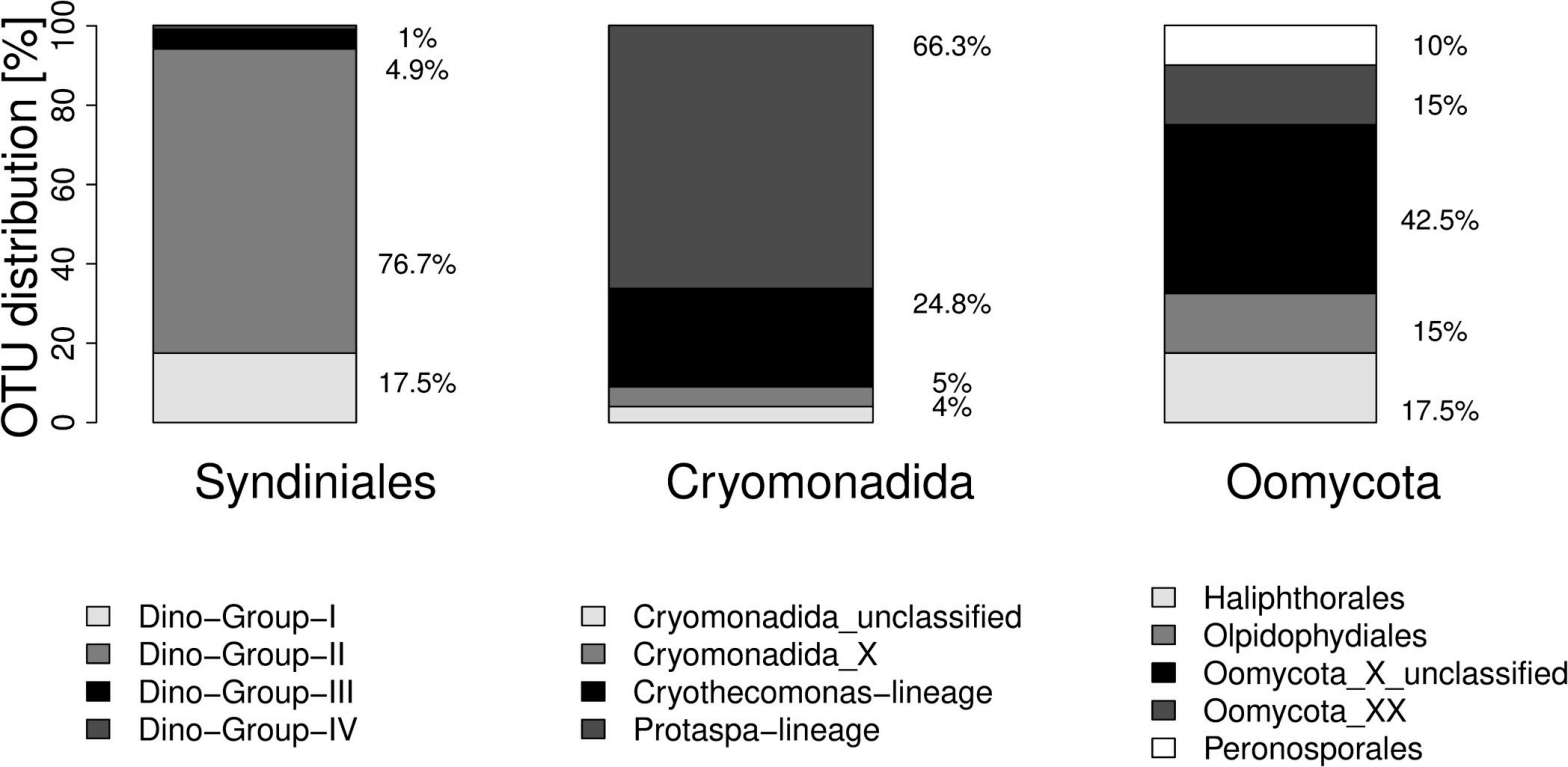
Distribution of OTUs in the different families of three different parasitoid taxa: Syndiniales, Cryomonadida and Oomycota.

The dinoflagellate phylum contributed to this **amount of OTUs** with more than 44% of all parasitoid OTUs (Table 1). All of these belonged to the exclusively parasitoid Syndiniales. We identified Syndiniales from four out of the five different Dino-Groups as they are named by the PR2 database (also known as Syndiniales-Groups) (Fig 1). Dino-Group-II, also known as Syndiniales-Group II, contributed the most OTUs (76.7%), followed by Group I (17.5%). Most OTUs of the Syndiniales could not be assigned further than family level. In all Dino-Groups only three genera of Syndiniales could be identified by PR2: *Syndinium*, *Euduboscquella* and *Hematodinium*. BLAST alignment revealed that eight out of ten OTUs found in Group III most probably belonged to the genus *Amoebophyra*.

**Fig 2:**
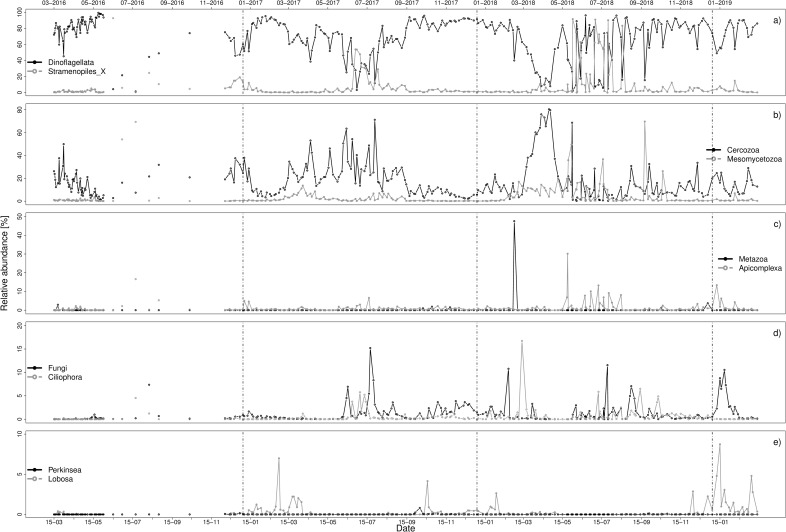
Relative parasitoid abundances [%] of parasitoid phyla, a) Dinoflagellata and Stramenopiles, b) Cercozoa and Mesomycetozoa, c) Metazoa and Apicomplexa, d) Fungi and Ciliophora, e) Lobosa and Perkinsea. Relative abundance is based on parasitoid taxa only. Note the different scaling of the axes. Vertical lines indicate turn of the years.

Syndiniales were also the biggest contributor in **relative read abundance** of all parasitoids. 22.5% of all dinoflagellate reads (including non-parasitoids) belonged to Syndiniales. With regard to the distribution of Syndiniales reads, 73% belonged to Group II, followed by Group I with 22%. Group III (3.9%) and Group IV (0.3%) was detected in lower read abundances. (S4 Table). Syndiniales, as the only dinoflagellate parasitoids, could be found throughout all years and seasons with declines in relative parasitoid abundance during spring as well as during July (Fig 2A).

The next biggest contributor (30%) in terms of OTU numbers was the phylum Cercozoa (Table 1). The phylum had its highest relative abundances during March and April, especially in 2018 as well as during summer in 2017 (Fig 2B). It included four classes, namely Endomyxa, Endomyxa-Phytomyxea, Filosa-Imbricatea and Filosa-Thecofilosea (Table 1), with known hosts such as green plants, brown algae and Stramenopiles including diatoms. Of these classes, Filosa-Thecofilosea and Filosa-Imbricatea had the highest relative parasitoid abundances. The order Cryomonadida was the most abundant out of all parasitoid Cercozoa taxa. 9% of the Cryomonadida OTUs could not be identified further (Fig 1). The highest number of OTUs belonged to the Protaspa lineage.

Parasitoid Stramenopiles made up over 10% of the parasitoid community (Table 1). While the phylum could be found in nearly all samples, the relative abundances of parasitoids were mostly low throughout the years, with peaks during summer months (Fig 2A). Highest relative parasitoid abundances were found in June 2016 (15-06-16), June/July 2017 and May to August 2018. We found two parasitoid Stramenopiles classes, namely *Pirsonia*-Clade (11 OTUs) and Oomycota (40 OTUs). Three families could be identified: Haliphthorales, Olpidophydiales and Peronosporales (Fig 1).

The phylum Mesomycetozoa included parasitoids of the class Ichthyosporea (Table 1), a group that can parasitize fish and crustaceans, which were mostly abundant during spring months (Fig 2B). In the phylum Fungi, parasitoid taxa in the classes Ascomycota and Chytridiomycota were found. Fungi were mainly present in June, July and August as well as during January 2019 (Fig 2D). Additional classes, some of which also included macro-parasite sequences in addition to parasitoids, were found mostly in low relative parasitoid abundances (Table 1 and Fig 2): Oligohymenophorea (Ciliophora), Apicomplexa_X (Apicomplexa), Nematoda (Metazoa), Tubulinea (Lobosa), Perkinsida (Perkinsea).

Each environmental parameter showed seasonal patterns as described below (S2 Fig, see also S1 Table), environmental conditions, therefore, were similar throughout all three years. Water temperature ranged from 1.9°C to 19.9°C depending on the season, while salinity ranged from 29.0 to 34.2. Secchi depth varied between 0.3 and 8.7 meter with several fluctuations. Silicate and nitrate both ranged from 0 to over 29 μmol L^-1^, highest concentrations were measured in winter and early spring months. Highest chlorophyll a concentrations were found in spring and summer with concentrations varying between 0.05 to 6.77 μg L^-1^. Daily sunshine duration varied greatly from day to day and ranged from 0 hours of sunshine to 15.6 hours.

Based on the CCA model, which included all 2790 OTUs, all implemented parameters except for tide were found to be significantly associated to the community structure: season (AIC = 2020.9, p = 0.005), total parasitoid occurrence (AIC = 2019.2, p = 0.005), temperature (AIC = 2018.4, p = 0.005), salinity (AIC = 2018.1, p = 0.005), silicate (AIC = 2017.7, p = 0.005), sunshine duration (AIC = 2017.5, p = 0.005), phosphate (AIC = 2017.4, p = 0.04), nitrate (AIC = 2017.5, p = 0.005) and Secchi depth (AIC = 2017.7, p = 0.005). In total, only 12.1% of inertia could be explained by all variables in full space. In restricted space the first axis explained 21.9% of the variance (2.7% in full space) and the second axis explained 20.4% (2.5% in full space). The CCA plot (S3 Fig) indicated that high parasitoid occurrences were not clearly correlated with any environmental parameter nor any specific season.

### Examples of known host-parasitoid systems at Helgoland

In the following, we display known host-parasitoid relationships, which were previously described in the literature and known to occur at Helgoland Roads, in order to check if the relationships can be found in the molecular dataset.

#### *Rhizosolenia imbricata*–*Olpidiopsis drebesii*

**Fig 3:**
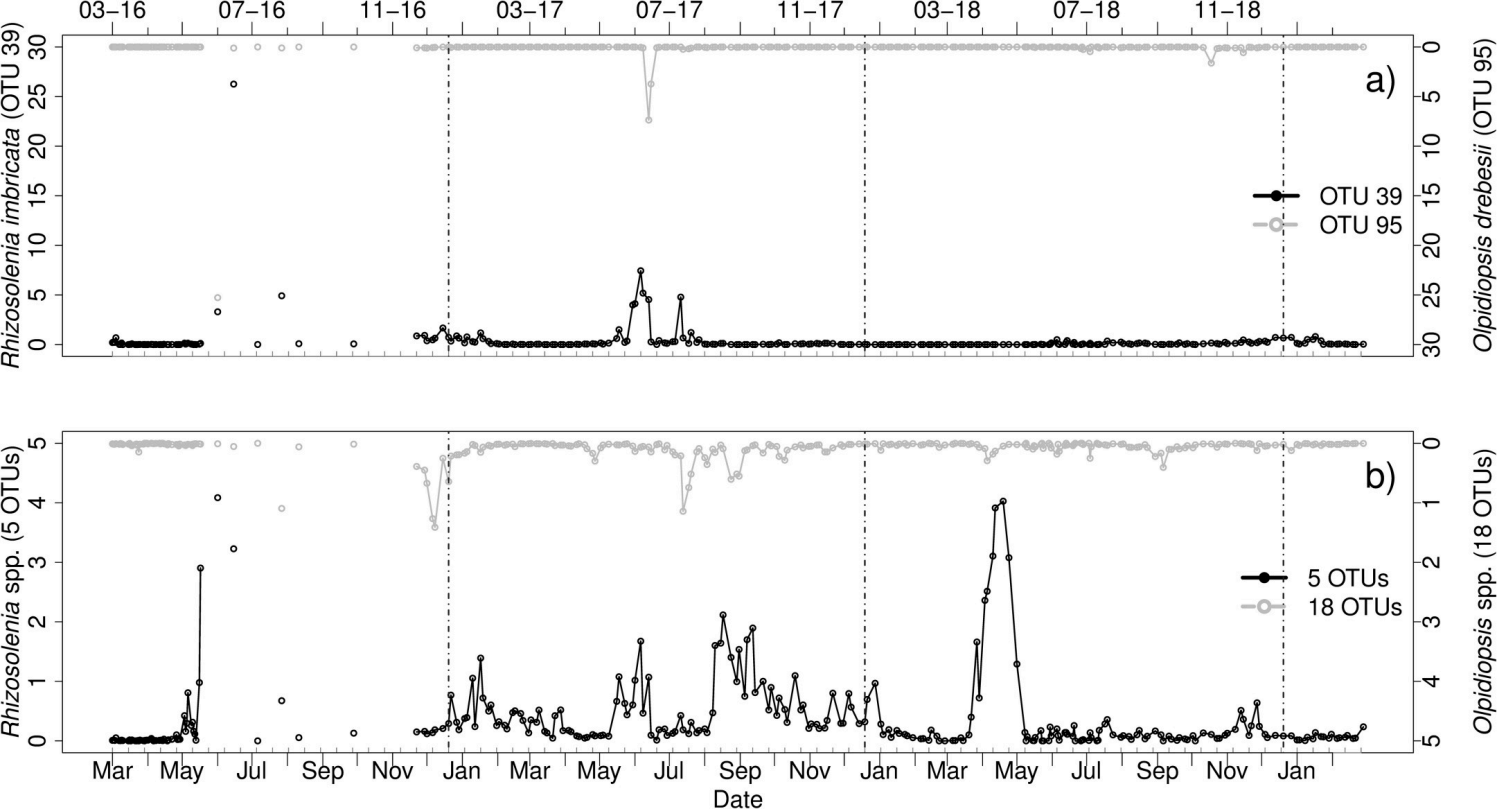
Relative abundances [%] of a) OTU 39 identified as *Rhizosolenia imbricata* (BLAST) and the parasitoid OTU 95 identified as *Olpidiopsis drebesii* (BLAST), and b) 5 OTUs identified as *Rhizosolenia* spp. (PR2) and 18 OTUs identified as *Olpidiopsis* spp. (BLAST) from March 2016 to March 2019. Vertical lines indicate turn of the years. Note the different scaling of the axes. Grey ticks on the x-axis indicate intervals of two weeks.

OTU 39 was identified as *Rhizosolenia imbricata* by BLAST alignment with a Score of 701 (PR2: *Rhizosolenia* sp.) and compared to occurrences of OTUs that were identified as Oomycota by PR2. BLAST alignment revealed 18 OTUs as potential *Olpidiopsis* species. Inter alia, OTU 95 was assigned to *Olpidiopsis drebesii*. Host and parasitoid OTU occurred every year (Fig 3A). Blooms of the host (OTU39) occurred in June 2016 and 2017. In June 2016 and 2017 the parasitoid reached peaks as well. In 2017, *Rhizosolenia imbricata* reached its peak on June 20^th^, while a peak of *O*. *drebesii* followed 7 days later, resembling our assumed case 1.

Several *Olpidiopsis* and *Rhizosolenia* OTUs that were identified to genus level (Fig 3B) revealed additional peaks of parasitoids infections. In August 2016, peaks of the host (*Rhizosolenia* spp. and OTU 39) and *Olpidiopsis* spp. occurred on the same day, which represents our case 2 (Fig 3A and 3B). The five OTUs of *Rhizosolenia* spp. revealed another bloom of the diatom in April and May 2018, however most peaks of that year were not closely linked to *Olpidiopsis* peaks.

#### *Pseudo-nitzschia pungens*–*Miracula helgolandica*

**Fig 4:**
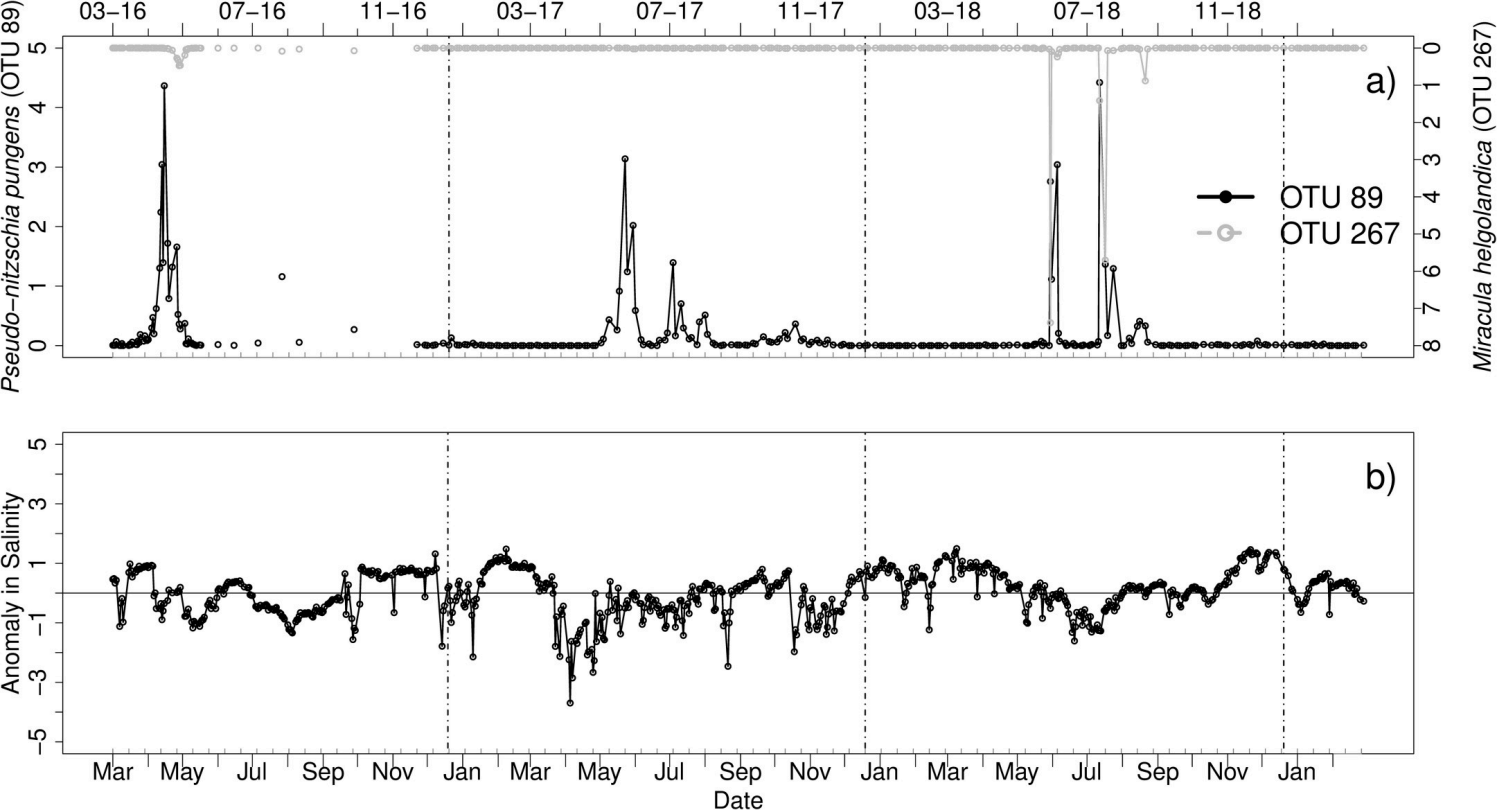
a) Relative abundances [%] of OTU 89 identified as *Pseudo-nitzschia pungens* (PR2) and the parasitoid OTU 267 identified as *Miracula helgolandica* (BLAST); b) anomaly in salinity from March 2016 to March 2019. Vertical lines indicate turn of the years. Note the different scaling of the axes. Grey ticks on the x-axis indicate intervals of two weeks.

OTU 89 (Fig 4A), which was identified as *Pseudo-nitzschia pungens* (PR2), was found to be co-occurring with the parasitoid OTU 267 *Miracula helgolandica* (identification by BLAST, Score: 678). *Pseudo-nitzschia pungens* usually occurred in the spring and summer months. It was blooming during April 2016 (26–04 to 29-04-16) and had further peaks in mid-May (06–05 to 12-05-16). In August, another peak was observed. In 2017, it was blooming in June and the highest peak was reached on June 06 (over 3%), followed by several smaller peaks in July (18–07 and 27-07-17) and August. The diatom was also blooming in summer 2018. It first peaked on June 13, followed by a second peak on June 19. The next big peak (over 4%) occurred in July (26-07-18). Afterwards a smaller peak followed on August 07.

The parasitoid OTU had its first occurrence during April and May 2016. For the rest of the year the parasitoid was either absent or occurrent without any distinct peak in abundance. In 2017, relative abundances were also low throughout the year and no distinct peak was detected. Several peaks, however, could be found in 2018, a first peak was reached in June (13-06-18) and a second peak appeared in July (31-07-18). The last smaller peak (below 1%) occurred in September (04-09-2018).

There were periods in 2016 and 2018, where host and parasitoid were closely aligned as defined for case 2. However, in 2017, large P. *pungens* blooms occurred without concurrent infection events. Comparison of host and parasitoid data with environmental conditions indicated that the absence of infections in 2017 coincided with a previous period of reduced salinity (Fig 4B).

#### *Coscinodiscus* sp.–*Lagenisma coscinodisci*

**Fig 5:**
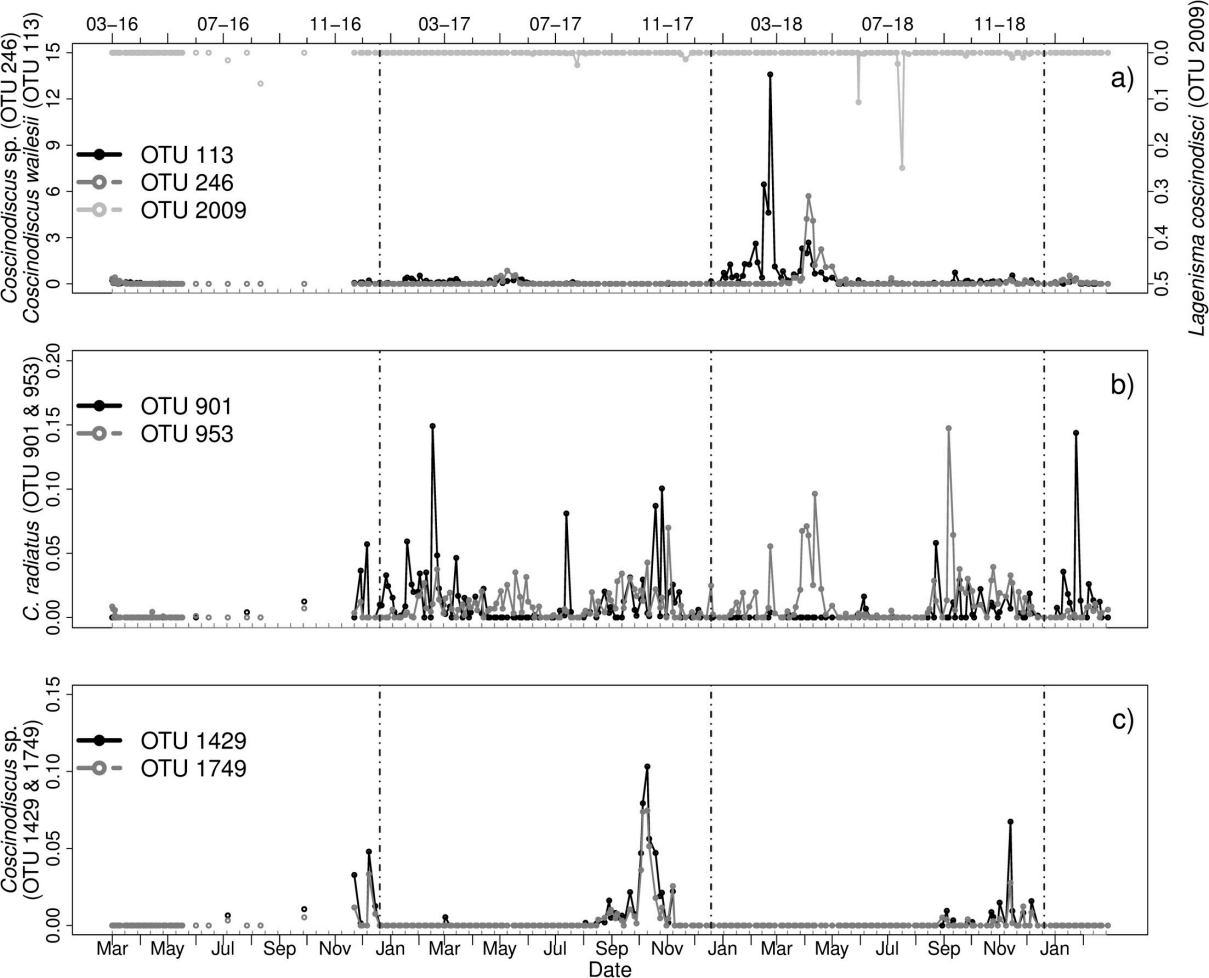
Relative abundances [%] of a) the parasitoid *Lagenisma coscinodisci* (BLAST, OTU 2009), the hosts *Coscinodiscus wailesii* (OTU 113), *Coscinodiscus* sp. (OTU 246); b) two potential *C*. *radiatus* sp. (OTU 901 and 953) and c) two *Coscinodiscus* sp. (OTU 1429 and 1749) from March 2016 to March 2019. Vertical lines indicate turn of the years. Note the different scaling of the axes. Grey ticks on the x-axis indicate intervals of two weeks.

Six OTUs were identified as *Coscinodiscus* sp., which included *Coscinodiscus wailesii* (OTU 113), two C. *radiatus* sp. (OTU 901 and 953) and three *Coscinodiscus* sp. which could not be further identified. OTU 2009 was identified as *Lagenisma coscinodisci* in BLAST (Score: 715). The parasitoid was found in 24 samples and in low relative abundances, as the maximum relative abundance was 0.25% on 31-07-18 (Fig 5A). Parasitoid read abundances peaked in August 2016 and 2017 (25-08-16, 08-08-17), and in June and July 2018 (13-06-18, 31-07-18). At these days no peaks of the host were found (Fig 5A, 5B and 5C).

All host OTUs occurred every year. In 2016 *Coscinodiscus wailesii* (OTU 113) was abundant in early spring and winter, in 2017 and 2018 in spring and summer and in winter 2018 until February 2019 (Fig 5A). It was blooming in February and March 2018 and had its biggest peaks during March 2018 (01-03-18: over 6%, 08-03-18: over 13%). A similar pattern was observed for OTU 246 (Fig 5A), which could only be identified up to genus level. Here the highest peak (over 5%) was found in April 2018. Two OTUs of C. *radiatus* (OTU 901 and 953) were only present in low relative abundances (below 0.02%) Both OTUs were continuously present during 2017 and 2018. OTU 901 had its biggest peaks in March 2017 and February 2019, OTU 953 in April and September 2018 (Fig 5B). The last two OTUs of *Coscinodiscus* sp. (1429, 1749) were also always below 0.2% in relative abundance and mostly present at the end of 2016, in autumn of 2017 and in winter 2018 (Fig 5C).

Co-occurrence as described by case 2 to the parasitoid was found for several of the host OTUs (OTU 113, 246, 953). However, no host peaks were aligned to peaks in the parasitoid. Instead these hosts were always low in abundant. A peak of OTU 901 might be linked to a parasitoid peak in 2018, which would resemble our case 1 (12 days).

#### *Guinardia* sp.—Cryomonadida and *Pirsonia* clade

**Fig 6:**
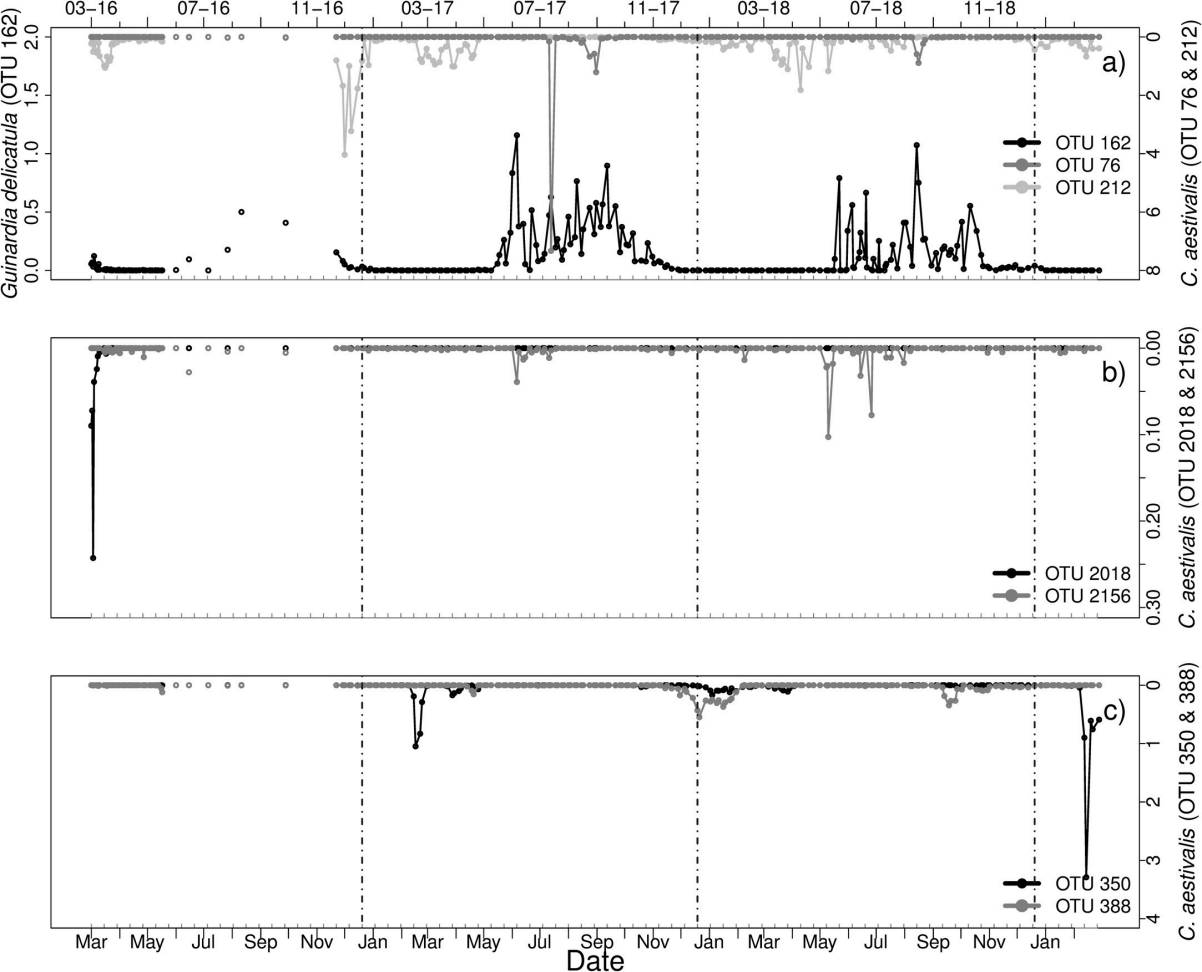
Relative abundances [%] of a) OTU 162 identified as *Guinardia delicatula*, and OTU 76 & 212 identified as *Cryothecomonas aestivalis* (BLAST), b) OTU 2018 & 2156 (*Cryothecomonas aestivalis*, BLAST) and c) OTU 350 & 388 (*Cryothecomonas aestivalis*, BLAST) from March 2016 to March 2019. Vertical lines indicate turn of the years. Note the different scaling of the axes. Grey ticks on the x-axis indicate intervals of two weeks.

**Fig 7:**
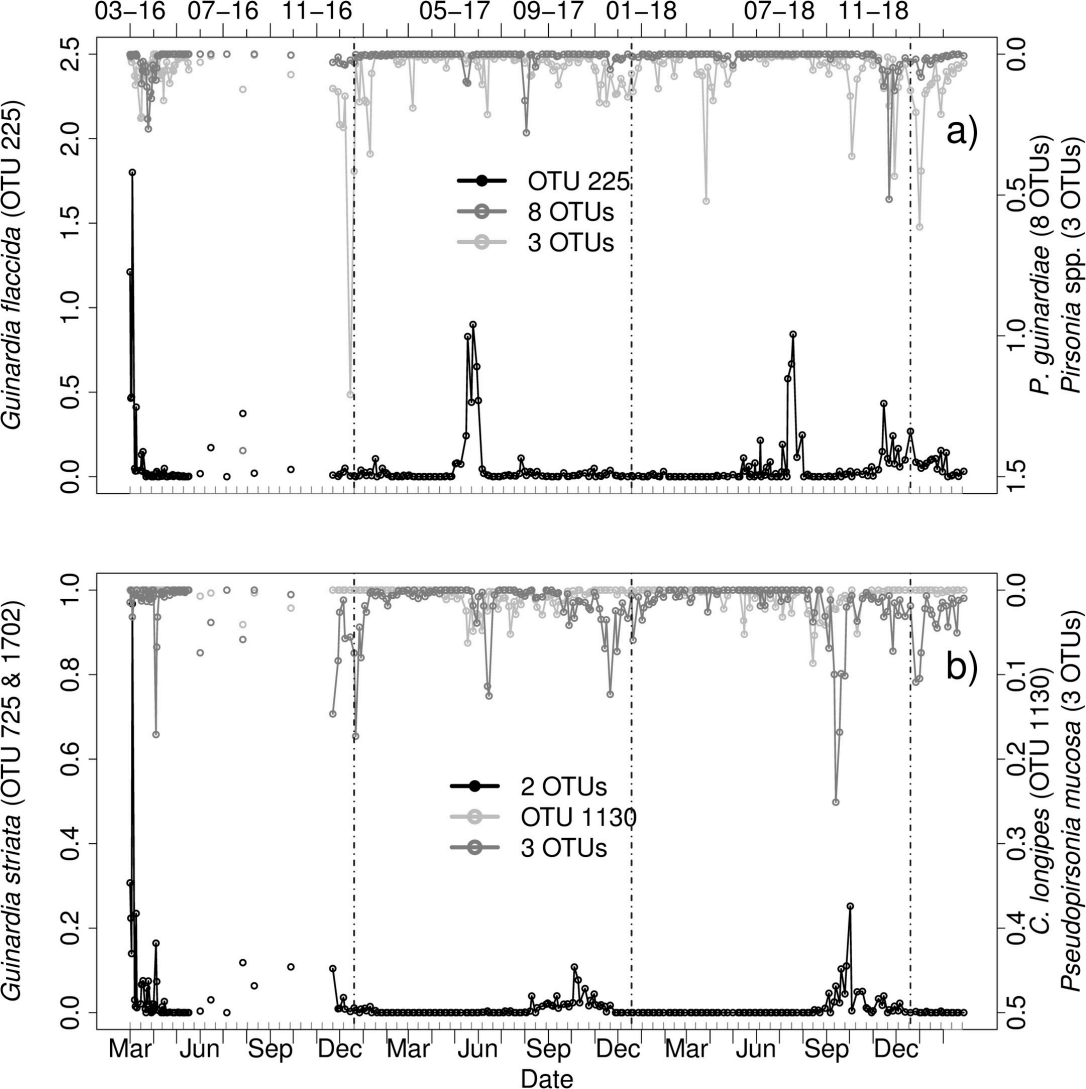
Relative abundances [%] of a) OTU 225 identified as *Guinardia flaccida* (PR2), *Pirsonia guinardiae* (8 OTUs) and *Pirsonia* spp. (3 OTUs), b) *Guinardia striata* (BLAST, OTU 725 and 1702), the parasitoid OTU 1130 identified as *Cryothecomonas longipes* (BLAST) and the parasitoid *Pseudopirsonia mucosa* (BLAST, 3 OTUs) from March 2016 to March 2019. Vertical lines indicate turn of the years. Note the different scaling of the axes. Grey ticks on the x-axis indicate intervals of two weeks.

Four OTUs of the diatom genus *Guinardia* (Figs 6 and 7) were found in the dataset: *Guinardia delicatula* (OTU 162, PR2), *Guinardia flaccida* (OTU 225, PR2). *Guinardia striata* (OTU 725, identified in BLAST, Score: 699) and OTU 1702 identified as *Guinardia striata* (BLAST, Score: 701). BLAST alignment of OTU 225 resulted in similar scores (701) for G. *flaccida* and G. *delicatula*, alignment of other OTUs confirmed the respective species as identified by PR2.

First, the known host-parasitoid system of G. *delicatula* and *Cryothecomonas aestivalis* was investigated. Out of all Cryomonadida OTUs (in total 101 OTUs) 27 OTUs were found as potential *Cryothecomonas aestivalis* (see S5 Table for PR2 and BLAST results of potential parasitoids). These OTUs were checked for co-occurrences to the host *G*. *delicatula*. The parasitoid was found in all samples. Most parasitoid OTUs were also present while the host was not present in the dataset (Fig 6).

The host G. *delicatula* was present in every year (Fig 6A). In spring 2016 G. *delicatula* was mainly present in March with a peak on March 18. During summer 2016 two peaks were detected in August (10–08 and 25–08). Furthermore, it was peaking on October 12 and in December 2016. In 2017 and 2018 G. *delicatula* was mostly occurring from May to December with several peaks and was blooming during June and July 2017 (e.g. between 15–06 to 20–06).

The association between *Guinardia delicatula* and C. *aestivalis* appeared to be complex and showed matches with different C. *aestivalis* OTUs throughout the sampling period as defined for case 2. For example for OTU 2018 in spring 2016, for OTU 2156 in June 2016, 2017 and 2018, and in July 2017, 2018 (Fig 6B), for OTU 76 in December 2016 and 2018 and for OTU 212 in summer 2017 (Fig 6A). For most OTUs the patterns hereby followed case 2, with simultaneous high abundances. Some parasitoid OTUs also showed high relative abundances after decline of the host OTU, such as OTU 76 in spring 2016, which indicates a relationship as described by case 1 in addition to co-occurrence as described by case 2. Additional peaks in parasitoid abundances did not match the occurrence of *G*. *delicatula*. These peaks, mainly occurring in late winter and early spring, included OTU 76 (January 2017, 2018 and February 2019), OTU 350 (February 2019) and OTU 388 in January 2018 (Fig 6A and 6C).

*Cryothecomonas aestivalis* is not the only parasitoid species known to infect *Guinardia* species. Additional *Cryothecomonas* species and *Pirsonia* clade were therefore also checked for co-occurrences with G. *delicatula* and other *Guinardia* OTUs (S5 Table). It is noteworthy that G. *flaccida* (OTU 225) had its highest relative abundances in March 2016 (Fig 7A) and occurred in low relative abundances without distinct peaks in February 2018, where other *Guinardia* OTUs were absent. BLAST alignment revealed eight out of eleven OTUs as potential *Pirsonia guinardiae*. Several co-occurrences (case 2) to their potential hosts were found throughout all years.

Furthermore, additional parasitoid OTUs were found to have similar occurrences compared to *Guinardia* OTUs (Fig 7A and 7B). These included for example OTU 1130, identified as *Cryothecomonas longipes* (BLAST, Score: 654) and three OTUs identified as *Pseudopirsonia sp*. and *P*. *muscosa*, respectively (PR2, verified in BLAST, S5 Table), indicative of possible additional infections as assumed by case 2 (Fig 7B).

### Examples of known host-parasitoid systems recorded at Helgoland for the first time

In addition to known host-parasitoid relationships the data set revealed some potential host-parasitoid associations which had not been described before for the area of Helgoland but are known from other areas in the world.

#### Dinoflagellates–Perkinsida

**Fig 8:**
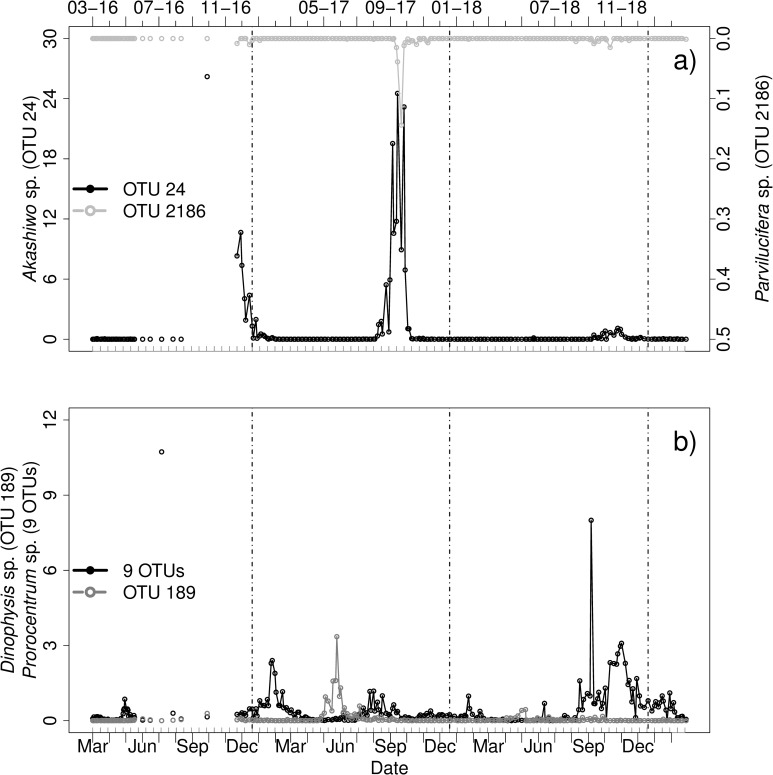
Relative abundances [%] of a) *Parvilucifera prorocentri* as identified by PR2 (OTU 2186) and *Akashiwo* sp. (OTU 24), b) *Prorocentrum* sp. (9 OTUs combined) and *Dinophysis* sp. (OTU 189) from March 2016 to March 2019. Vertical lines indicate turn of the years. Note the different scaling of the axes. Grey ticks on the x-axis indicate intervals of two weeks.

We found one OTU belonging to the Perkinsida, which was identified as *Parvilucifera* sp. (PR2: *Parvilucifera prorocentri*). In BLAST it was identified as another Perkinsida species *Dinovorax pyriformis* (Score 516). As Perkinsida are known to infect dinoflagellates, the occurrence of this OTU (Fig 8A) was compared to the occurrence of known host species as well as additional dinoflagellates. *Parvilucifera prorocentri* peaked in September and October 2017, as well as in October 2018, with its highest peak occurring in 2017 on October 5. The two known host genera *Prorocentrum* sp. and *Dinophysis* sp. did not show a clear association with P. *prorocentri* as no peaks were detected in October 2017(Fig 8B). However, corresponding to case 1, a time delay of seven days was observed between the maximum occurrence of *Akashiwo sp*., which was blooming in autumn 2017, and the parasitoid (Fig 8A).

#### *Eucampia zodiacus*–Cercozoa

As the diatom *Eucampia zodiacus* is known to be infected by different species, the dataset was used to check for these potential parasitoids. Additionally, a parasitic infection was visible in several microscopic images (retrieved from planktonnet.awi.de, S4 Fig). The infections were visible in live cells from July as well as August 2017.

**Fig 9:**
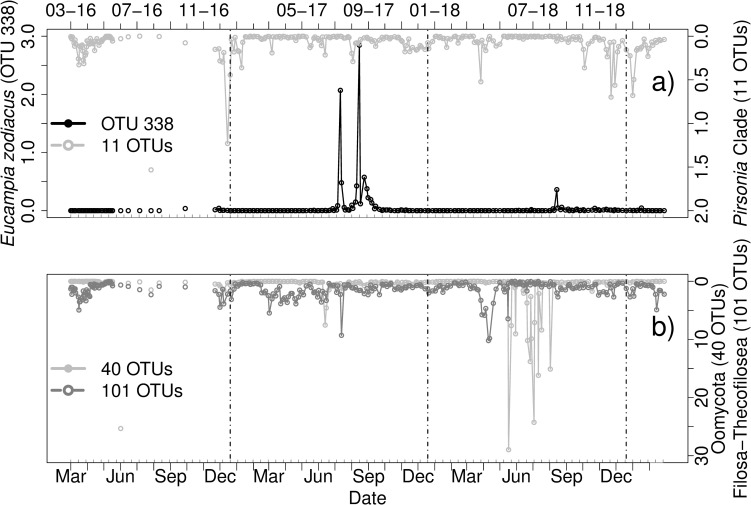
Relative abundances [%] of a) OTU 338 identified as *Eucampia* sp. (PR2) and the parasitoid taxa *Pirsonia* Clade (11 OTUs), b) Oomycota (40 OTUs) and Filosa-Thecofilosea (101 OTUs) from March 2016 to March 2019. Vertical lines indicate turn of the years. Note the different scaling of the axes. Grey ticks on the x-axis indicate intervals of two weeks.

In our dataset *Eucampia zodiacus* was mostly present in summer 2017. The diatom host *Eucampia sp*. had a first peak (over 2%) on 25-07-17, a second bigger peak on 29-08-17 (over 2.8%) and a third smaller peak (over 0.5%) on 07-09-17 (Fig 9A). *Pirsonia*-Clade, which includes taxa that can infect *Eucampia zodiacus*, as well as Oomycota and Filosa-Thecofilosea abundances were compared to the occurrence of this host (Fig 9B). Several co-occurrences (case 2) and alternating associations (case 1) between the host and different parasitoids were found, including inter alia OTU 212 identified as Cryothecomonas aestivalis (BLAST Score: 673) and several OTUs belonging to *Pirsonia*-Clade (see S6 Table for PR2 and BLAST results of potential parasitoids).

#### Syndiniales genera–Crustacea & Tintinnida

**Fig 10:**
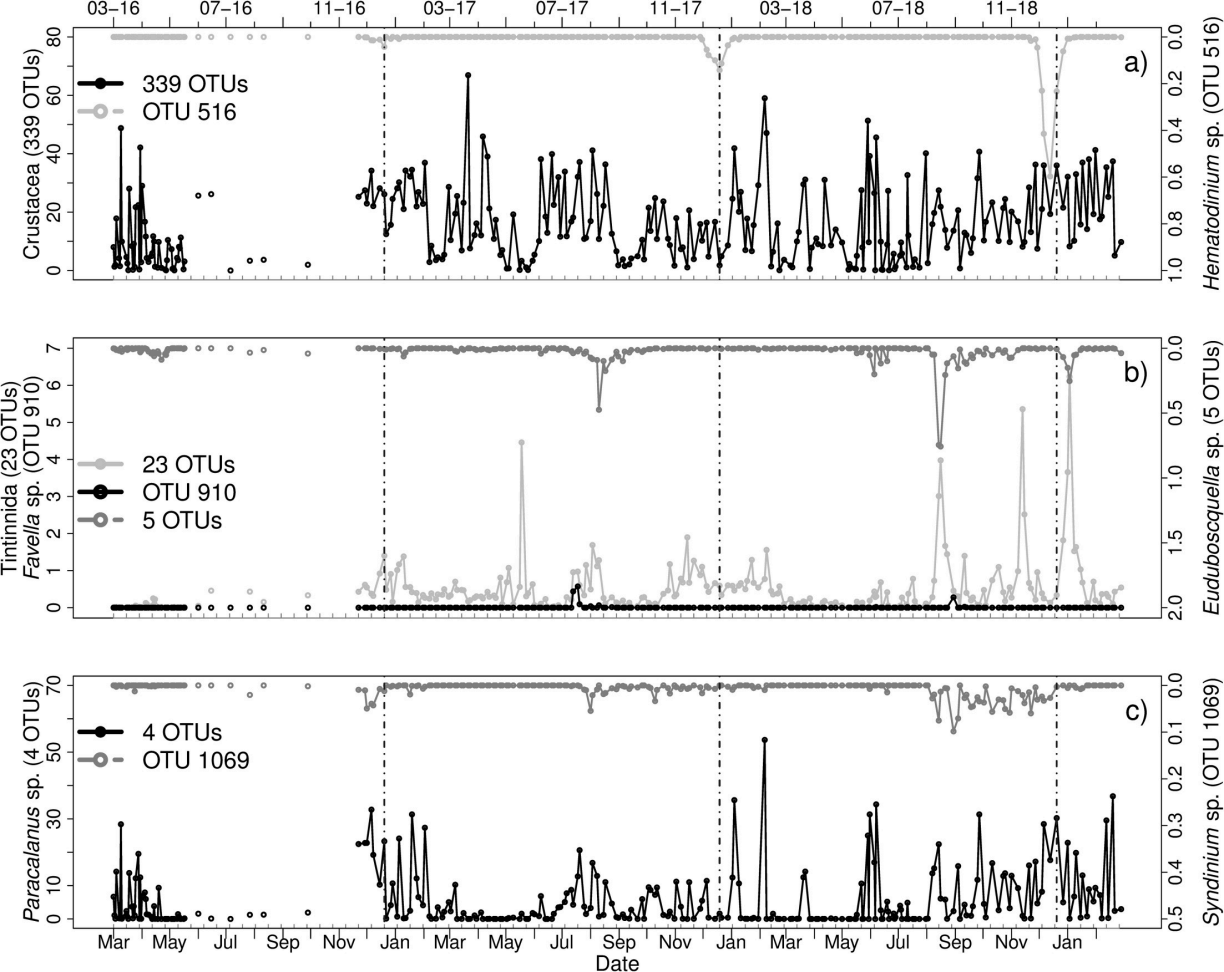
Relative abundances [%] of a) 339 OTUs identified as Crustacea (PR2) and the parasitoid *Hematodinium* sp. (OTU 516, Syndiniales, PR2), b) Tintinnida (23 OTUs), *Favella* sp. (OTU 910, PR2) and the parasitoid *Euduboscquella* sp. (5 OTUs, Syndiniales, PR2) and c) *Paracalanus* sp. (4 OTUs, PR2) and the parasitoid *Syndinium* sp. (OTU 1069, Syndiniales, PR2) from March 2016 to March 2019. Vertical lines indicate turn of the years. Note the different scaling of the axes. Grey ticks on the x-axis indicate intervals of two weeks.

Three different genera of Syndiniales (*Hematodinium* sp., *Euduboscquella* sp. and *Syndinium* sp.) could be identified and were compared to potential host OTUs. For *Hematodinium* sp. two peaks in relative abundance were found (02-01-18 and 27-12-18). The peak at the end of 2018 was co-occurring with high relative abundances of Crustacea (Fig 10A). This high abundance was mainly caused by 4 OTUs (identification by PR2): *Paracalanus* sp. (OTU 1), *Temora* sp. (OTU 2), unclassified Maxillopoda (OTU 27) and *Tachidius* sp. (OTU 38).

*Favella* sp. a known host of *Euduboscquella* sp. had its biggest peaks in occurrence from 27-07-2017 to 03-08-2017 and in September 2018.The parasitoid occurred during all years with several peaks in abundance (Fig 10B). On August 24 2017, *Euduboscquella* sp. reached a peak in relative abundance of over 0.4%, where the host was also present. In 2018, the peak of the parasitoid occurred in absence of the host OTU. Some of the parasitoid peaks were also co-occurring with other Tintinnida.

*Syndinium* sp. also had several peaks in abundance, for example in December 2016, in August 2017 and from August to December 2018 (Fig 10C). Other peaks of *Syndinium* sp. were also co-occurring with *Paracalanus* sp. during all years.

### Identification of potentially new host-parasitoid systems

Identification of new potential systems proved to be very difficult, since known systems as described in the previous paragraphs did not show consistent dynamics (see also Table 2). Thus using population dynamical information to identify other pairs based just on temporal dynamics of known interactions is not a promising venue. Particularly, the high diversity of potential parasitoid and hosts leaves a high level of speculation even on co-occurring OTUs.

**Table 2:** Overview of parasitoid dynamics.

System	Observed dynamics	Observed Time delay
*Rhizosolenia imbricata*–*Olpidiopsis drebesii*	Case 1 and 2	7 days
*Pseudo-nitzschia pungens–Miracula helgolandica*	Case 2	
*Coscinodiscus* sp.–*Lagenisma coscinodisci*	Case 1 and 2	12 days
*Guinardia* sp.—Cryomonadida and *Pirsonia* clade	Case 1 and 2	up to several days
*Akashiwo* sp.—*Parvilucifera prorocentri*	Case 1	7 days
*Eucampia zodiacus*—Cercozoa	Case 1 and 2	2 to 7 days
Syndiniales genera–Crustacea & Tintinnida	Case 2	

## Discussion

### Identifying parasitoids

A wide diversity of parasitoids, which are known to be associated with a suite of different hosts, could be identified at Helgoland Roads. At the same time, the variability in the dynamics of known host-parasitoid pairs was considerable with many instances. For example, either hosts or parasitoids occurred separately, they showed some sort of Lotka-Volterra type alternating cycles or they co-occurred. Hence, our goal to use the dynamics of known pairs to identify potential thus far unknown host-parasitoid sets was essentially doomed from the start.

Due to the high abundances in parasitoids and the number of species present at different times of the year, infections can essentially occur throughout the year. For example, some parasitoid phyla were found as isolated events in a specific year such as Fungi, Apicomplexa, Metazoa and Perkinsea. Other taxa were present nearly throughout the whole sampling periods (e.g. Syndiniales and Cercozoa). Importantly, many trophic levels from primary producers to secondary consumers can potentially be affected. The potential hosts range from diatoms (e.g. Oomycota) to fish (e.g. Ichthyosporea) depending on the parasitoid species or group.

Highest abundances were found for the parasitoid dinoflagellates from the Syndiniales class. However, it was impossible to find clear correlations to potential hosts. The high read abundances are in accordance with generally high read abundances of dinoflagellates at Helgoland. Moreover, since it has been known that Syndiniales have low chromosome numbers compared to Dinophyceae [87], we can conclude that the high abundances are not caused by potential sequencing biases. Besides different Dino-Groups that cannot be further identified, we found known genera such as *Euduboscquella*, *Syndinium*, and *Hematodinium* present in our dataset. Among others, the three genera are known to infect tintinnid ciliates [88, 89], and crustaceans such as calanoid copepods, crabs and lobsters [20, 65, 90], respectively.

There have been suggestions about Syndiniales not always having a clear host-specificity [33]. For known genera, such as the parasitoid *Amoebophyra*, it has been shown that even though hosts were killed, other potential hosts in the same water mass were not declining even though a large number of dinospores were released [26]. The dinospores, that are released in large numbers, are short-lived and so far, they are known to complete their life cycle in a few days [33]. The high abundances are in accordance with other environmental studies, where Syndiniales showed high abundances especially in pico- and nanoplankton size fractions [91, 92], also in Antarctic winter [31]. It has been suggested that the free-living dinospores are mostly picoplanktonic, while an increase of abundances in bigger size fractions represent the parasitoids in their infectious stage in their host cell [22]. The fact that Syndiniales sequences can be found in high diversity throughout the year, could be explained in a number of scenarios. For instance it might be that they are only facultatively parasitoid, that production of new spores is either constant or that additional, so far unknown, life cycle stages exist [33], but this will require further investigation.

It needs to be noted that a majority of parasitoids is still poorly investigated on the molecular level as well. DNA sequences on species level are scarce for some groups including host taxa, which implies that protistan parasitoids can be even more diverse than known today [20]. As discussed before [47], there are several methodological issues such as choice of target region and database that influence identification. For example, comparison of V4 and V9 sequencing revealed differences in community diversity and weaknesses regarding identification of specific taxonomic groups like Chlorophyta, Ciliates or full eukaryotic communities [93–95]. The combination of different primer pairs and addition of mock communities to the analysis to decrease these weaknesses were suggested so far [95, 96]. Additionally, the V4 region has been found to have a bigger taxonomic resolution compared to the V9 region [97, 98]. The use of different pipelines results in not-reproducible outputs and differences in assigned taxa as it has been shown for diatoms [99], which makes it important to include all potential parameters in the methodology. While tuning on parameters might increase coverage of community composition, we focused on using a strict parameter set and a high confidence cut-off of annotation aiming for a high reliability. Furthermore, comparison of different parameter sets revealed that our main findings are pretty robust against changes in the parameter values. The drawback in molecular identification is also noticeable for the whole plankton community as identification not only on species level is scarce and assignment of trophic modes therefore is not possible for big parts of the community. It is also evident when comparing identification results from the PR2 database and BLAST alignment, where contradictory results occurred even for potential hosts, not only on species level (e.g. OTU 225: *Guinardia flaccida* or G. *delicatula*), but also when comparing higher taxonomic levels. For example, while PR2 could identify OTU 725 only up to family level (Radial-centric-basal-Coscinodiscophyceae), through BLAST alignment it could be identified as *Guinardia striata* (Score: 699). Furthermore, PR2 identified several OTUs as belonging to the parasitoid *Protaspa*-lineage, whereas BLAST results indicate that the OTUs belong to *Cryothecomonas longipes*. Hereby, the BLAST results could be supported by construction of a maximum likelihood tree of the Cryomonadida OTUs (S5 Fig) in MEGA X [100] by use of Tamura-Nei model [101].

With respect to the influence of environmental conditions on parasitoids occurrences and infections, correlations with temperature are known. For example, for *Cryothecomonas aestivalis* infecting *Guinardia delicatula* on the New England Shelf, the highest infection rates only occurred at water temperatures of above 4°C. The host on the other hand was blooming at a greater range of temperature below and above 4°C [27]. This indicates that environmental conditions influence the presence of parasitoid and the opportunity for infections and that host and parasitoid are not necessarily perfectly synchronized in terms of their environmental tolerances. In our study, we cannot confirm this phenomenon. The host *G*. *delicatula* (OTU 162) was only found to be abundant, when the water temperature was above 5°C, while *C*. *aestivalis* was present at all temperatures, that ranged from 2.7°C to 19.7°C. However, another host-parasitoid system indicates influence of the environmental conditions to development of the parasitoid. *Miracula helgolandica* was described and isolated from *P*. *pungens* at Helgoland [42]. While the host was present in high abundances during 2017, the parasitoid did not notably peak in abundance. Highest peak abundances in the host were found for temperatures above 10°C (up to 19.7°C) and the parasitoid occurred at similar temperature ranges except 2017. Anomalies in salinity might have influenced the availability of *Miracula* instead. Additionally, differences in timing and life cycle developments can be influential, especially since *P*. *pungens* occurred in short time corridors throughout the sampling period.

### Recognizing known host-parasitoid systems using NGS

It was possible to find co-occurrences of known host-parasitoid systems at Helgoland such as host *Rhizosolenia imbricata* which was infected by *Olpidiopsis drebesii* [42] and *Pseudo-nitzschia pungens*, which is known to be infected by *Miracula helgolandica* [24, 42]. These parasitoids have been described as new species at Helgoland and since then could be observed frequently. The parasitoid *Lagenisma coscinodisci* has been observed in detail in the past [40, 41, 73, 74] and was found in our dataset, however, *Lagenisma coscinodisci* relative abundances were generally low throughout the sampling period.

Identification of other known systems turned out to be more complex with respect to host specificity and therefore their potential contribution to the seasonal dynamics within the plankton at Helgoland Roads. An example is presented by the genus *Guinardia*. The three species known to be present at Helgoland Roads, are all known to be parasitized by the parasitoids *Cryothecomonas*, *Pirsonia* and *Pseudopirsonia* [15, 18, 19, 23–25, 27]. In our study, parasitoid occurrences were overlapping with different species. For example, the peak in abundance of *Guinardia flaccida* during February 2017 and December 2018 was matching with several different parasitoid taxa such as C. *aestivalis*, C. *longipes*, *Pseudopirsonia muscosa* and *Pirsonia guinardiae*. This suggests that coincident infections of the identified *Cryothecomonas* OTUs and *Pirsonia* took place in this taxon. While this indicates that simultaneous infections by different parasitoids are likely, the loss or lack of host specificity of certain parasitoids also increase the complexity of the system.

A new potential host-parasitoid system for Helgoland was found for *Parvilucifera prorocentri* and an OTU of the genus *Akashiwo*. The parasitoid is known to have dinoflagellate hosts such as *Dinophysis* sp. and *Prorocentrum* sp. [86]. However, comparison of the occurrences showed, that these known host species were not associated with the parasitoid in our study. Our first assumption was loosely based on the Lotka-Volterra model, defined as periodic fluctuations with a certain time lag [30]. For *Akashiwo sp*. this assumption in predator-prey dynamics was observed. The example hints at the potential of parasitoids for controlling plankton blooms and their consequences for the food web. However, linking these rapid changes in host abundance to further potential host-parasitoid associations is not easy. So far, *Parvilucifera* infections of the dinoflagellate *Akashiwo sanguinea* were only observed in Masan Bay, Korea in April 2015 [102].

After comparison with other host-parasitoid systems, it was hard to detect alternating associations with time lags between host and parasitoid in addition to the *Akashiwo*–*P*. *prorocentri* system. For June 2017 we could find a delay of several days between the peaks of host *Rhizosolenia imbricata* and parasitoid *Olpidiopsis drebesii*, while this delay was not visible during other co-occurrences. For *Guinardia delicatula* and OTU 76 also both cases could be suggested, however simultaneous appearances, and therefore current infections (case 2), were mostly observed for all other C. *aestivalis* OTUs.

In addition to inspection of sequencing data, we could find microscopic evidence for a parasitic infection of the diatom *Eucampia zodiacus*. Thus far, known parasitoids for *Eucampia* are *Pirsonia* sp. like *Pirsonia eucampiae* and *Pirsonia formosa* [15] or *Paulsenella kornmannii* [103]. While *P*. *eucampiae* and *P*. *kornmannii* were not found in the sequencing dataset, *P*. *formosa* was identified as a potential parasitoid species. However, this OTU was present during times, where *Eucampia zodiacus* was not detected and BLAST identification was inconclusive. Therefore, this is an indication that additional parasitoids are infecting *Eucampia*, which are still unknown. One potential parasitoid might be OTU 212, which peaked in abundance shortly after *Eucampia*. If looking at the dataset, several additional potential parasitoids were occurring simultaneously. However, some of these potential parasitoids are not likely infecting *Eucampia*. Some co-occurrences might happen by chance, since other potential hosts could be present at the same time. For example, several OTUs were identified as *Protaspa grandis*, which is bigger in size than the parasitoid which was found by microscopy. This species is known to reach sizes from 32.5–55.0 mm in length and 20.0–35.0 mm width [104]. In addition, visual comparison of known parasitoids indicates that some OTUs are unlikely to be a potential parasitoid of *Eucampia*. One example is *Olpidiopsis drebesii*, which can be excluded, if we inspect and compare the morphology as described for infections in *Rhizosolenia imbricata* [42].

### Is identification of unknown host-parasitoid systems possible using NGS data?

In regard to high temporal resolution sequencing studies, previously observed host-parasitoid systems might not follow the expected dynamics. Since other co-occurrences were mostly found to be happening simultaneously and without delay between host and parasitoid and since DNA of the parasitoid should be able to be detected from its host, a match in peak abundance between host and parasitoid hints towards a current infection. In addition, for both–host and parasitoid the environmental conditions need to be favourable for an infection to occur [105, 106]. Additional shifts in the physico-chemical environment, pertinently, in temperature and differences in thermal tolerances, in addition to changes in timing of occurrence, might cause the decoupling of existing host-parasitoid systems and the development of new relationships, increasing of infection rates and shifts in local food webs [107, 108]. In case of short-lived infections, long gaps in time between sampling might reduce recognition of this phenomenon. However, this is unlikely here due to our high sampling frequency in sampling phase 1 and 3, even though an even higher sampling frequency might cover short-lived infections that might occur within one day. Furthermore, knowledge of survival of parasitoids without their host and the life cycle of free-living states is scarce for most new described parasitoids since they are hard to detect with microscopy and mostly based on culturing experiments. While it is not possible to distinguish different stages in sequencing, the presence of the parasitoid can still be detected with this method. Another issue is the potential mismatch in timing of host and parasitoid occurrences and the influence of environmental conditions on the life cycles. Given the complexity of the life cycles, the diversity of parasitoid-host relationships within the system as well as their interaction with environmental conditions, it might be too simple to expect a typical Lotka-Volterra type dynamic for identifying host-parasitoid systems, since typical and clear parasitoid-host phenomenon as described by Alves-de-Souza et al. [29] might be the exception rather than the rule.

The high dynamics of parasitoid occurrence and the variability in infection dynamics made it hard to detect host-parasitoid relationships using our sequencing dataset. Reasons for this might be the possibility of infections by several parasitoids either simultaneously or at different times, the fact that parasitoids could be plurivorous and that free-living stages cannot be distinguished by sequencing.

## Conclusions

Our study is, to our knowledge the first, investigating multiple host-parasitoid systems and dynamics of parasitoids over a number of years. We have shown the high prevalence of parasitoids at Helgoland in high temporal resolution. The flexibility in parasitoid infections might have a big impact to the seasonal dynamics of the plankton community at Helgoland Roads. This highly detailed study also revealed several host-parasitoid systems with different temporal patterns such as simultaneous appearances, alternating cycles (with or without regular lags) and persistent parasitoid occurrence (Syndiniales). Potential systems that have been mentioned here, might be verified by microscopic and further molecular analysis such as newly developed fluorescence in situ hybridization probes. To adequately capture the complexity and high variability of host-parasitoid interactions and dynamics, further research on the dataset are necessary, especially since it was impossible to identify new systems with NGS alone.

Due to the high abundances, broad temporal occurrence patterns and their considerable diversity, we consider there to be a high likelihood of parasitoid infections on different components of the food web. The high diversity also shows that effects on the whole food web are likely, since parasitoids found are known to infect hosts of all trophic levels. While a high chance of parasitic infections adversely affects single hosts throughout the food web, this phenomenon might in contrast positively affect the whole community and the resilience of the system. The infection of one component of the food web can help the growth of other populations, which would not have evolved with the other population present. This in turn makes this topic even more relevant for future investigations on food web dynamics.

## Supporting information

S1 Fig Relative abundances [%] of parasitoids and non-parasitoid OTUs. Non-parasitoid OTUs include all remaining OTUs, that were not identified as Parasitoids; Vertical lines indicate turn of the years.
(TIF)

S2 Fig Overview of environmental conditions, a) water temperature, Secchi depth, b) Salinity, Tide, c) Silicate, Nitrate, d) Chlorophyll a, Sunshine duration from March 2016 to March 2019. Vertical lines indicate turn of the years. Note the different scaling of the axes.
(TIF)

S3 Fig Canonical Correspondence Analysis (CCA) of the samples (grey asterisks with sampling date) including significant parameters in black: Temperature (temp), salinity (sal), silicate (SiO4), nitrate (NO3), sunshine duration (sun), total parasitoid occurrence (parasitoids), seasons (spring, summer, autumn, winter) and tide (low tide, high tide). 12.2% of total inertia could be explained by all variables in full space, in restricted space CCA1 explained 23.8% of the variance and CCA2 explained 20.9%.
(TIF)

S4 Fig Live cells of the centric diatom Eucampia zodiacus collected at Helgoland Roads, a) without parasitic infection (3rd August 2017), b)-d) with parasitic infection (b) 27th July 2017, c-d) 29th August 2017). Figures retrieved from planktonnet.awi.de.
(TIF)

S5 Fig Maximum likelihood tree of Cryomonadida OTUs. The evolutionary history was inferred by using the Maximum Likelihood method and Tamura-Nei model [101]. The tree with the highest log likelihood (-3807.40) is shown. Initial tree(s) for the heuristic search were obtained automatically by applying Neighbor-Join and BioNJ algorithms to a matrix of pairwise distances estimated using the Tamura-Nei model, and then selecting the topology with superior log likelihood value. This analysis involved 101 nucleotide sequences. There were a total of 397 positions in the final dataset. Evolutionary analyses were conducted in MEGA X [100].
(TIF)

S1 Table Sampling information and environmental parameters. (XLSX)

S2 Table Sequencing statistics. Raw: raw sequences after demultiplexing; Trimmed: remaining sequences after 3’-quality trimming; Assembled: remaining sequences after paired-end merging; Primer filtered: remaining sequences after removing primers; Feature filtered: remaining sequences after filtering for length; Sample derep: amount of unique sequences; Chimera filtered: remaining unique sequences after chimera removal; Final rerep: remaining sequences if we would rereplicate the sequences; Avg length: average length of each sequence in the sample.
(XLSX)

S3 Table Relative parasitoid abundances of parasitoid OTUs. Relative abundance is based on parasitoid taxa only.
(XLSX)

S4 Table Proportional distribution of Syndiniales clades detected over the whole timeframe. (XLSX)

S5 Table Relative abundances [%] of potential parasitoids of *Guinardia* sp. after manual identification. (XLSX)

S6 Table Relative abundances [%] of potential parasitoids of *Eucampia* sp. after manual identification. (XLSX)

S1 File Additional information on bioinformatic pipeline and analysis. (DOCX)
