# Supplementary figures and images for: Host-parasitoid associations in marine planktonic time series: Can metabarcoding help reveal them?
Source: PLoS One. 2021 Jan 7;16(1):e0244817. doi: 10.1371/journal.pone.0244817 (PMC7790432; doi:10.1371/journal.pone.0244817)

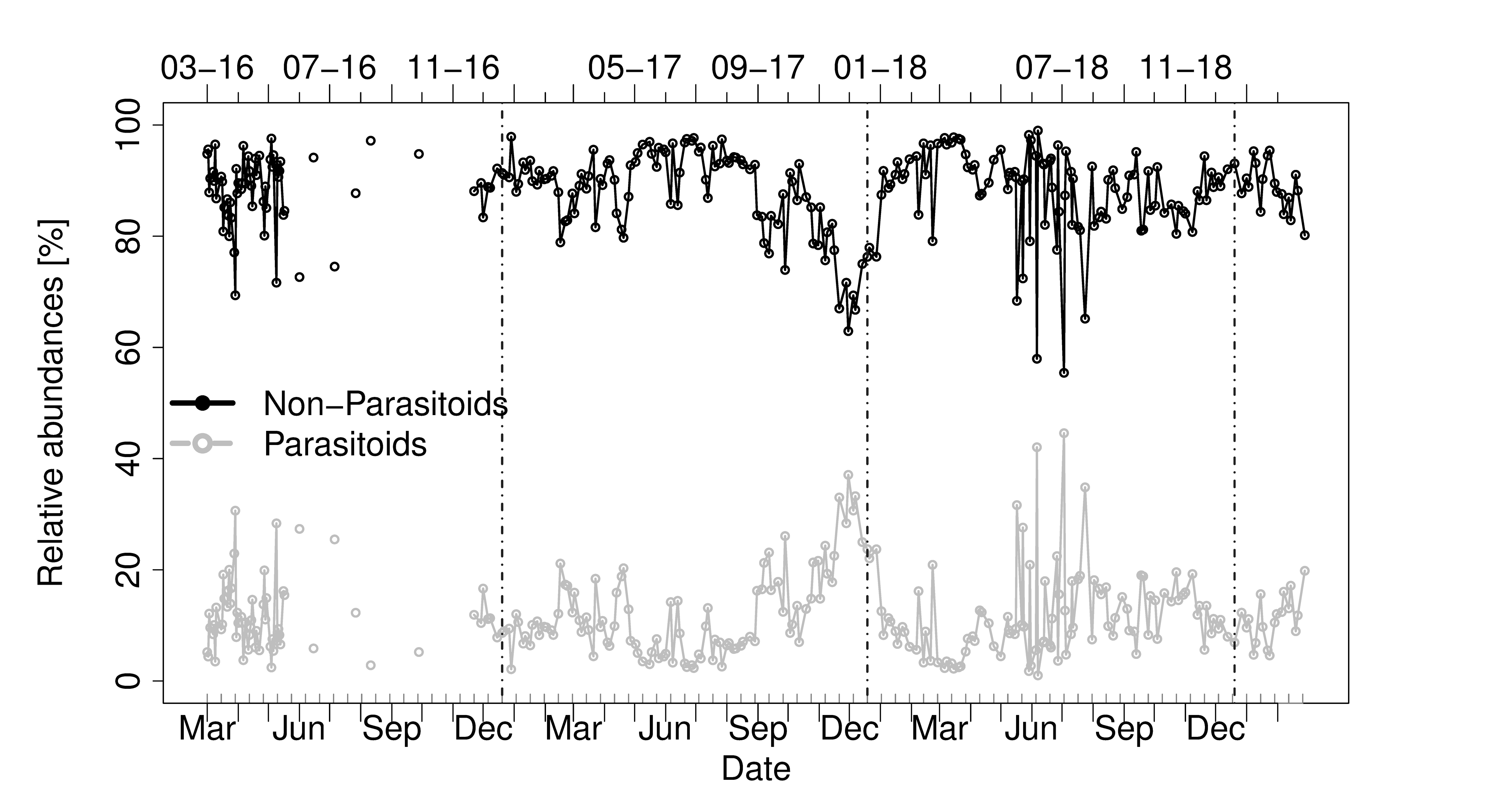

Supplement: S1 Fig — Non-parasitoid OTUs include all remaining OTUs, that were not identified as Parasitoids; Vertical lines indicate turn of the years. (TIF) [file pone.0244817.s001.tif]

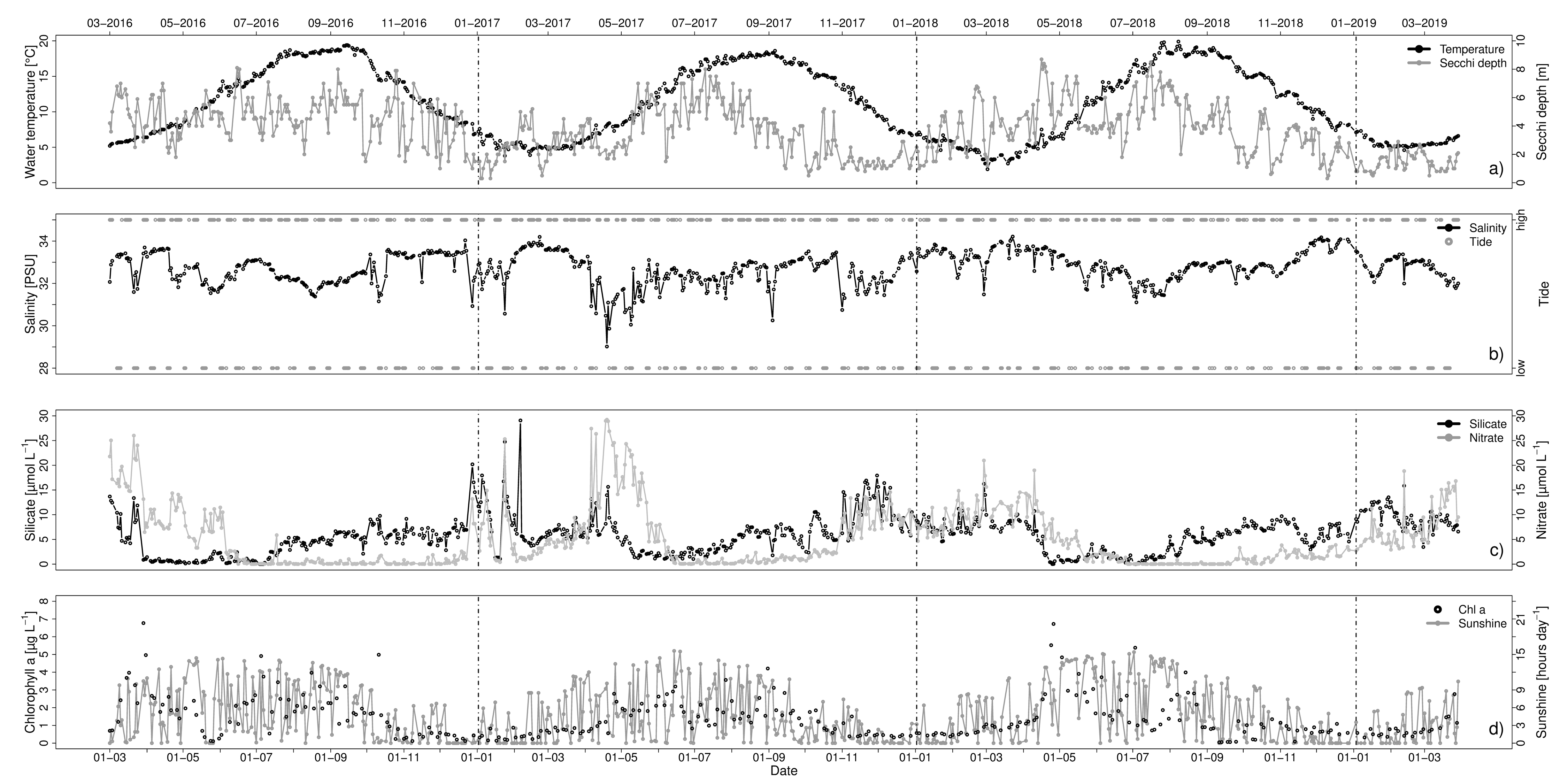

Supplement: S2 Fig — Overview of environmental conditions, a) water temperature, Secchi depth, b) Salinity, Tide, c) Silicate, Nitrate, d) Chlorophyll a, Sunshine duration from March 2016 to March 2019. Vertical lines indicate turn of the years. Note the different scaling of the axes. (TIF) [file pone.0244817.s002.tif]

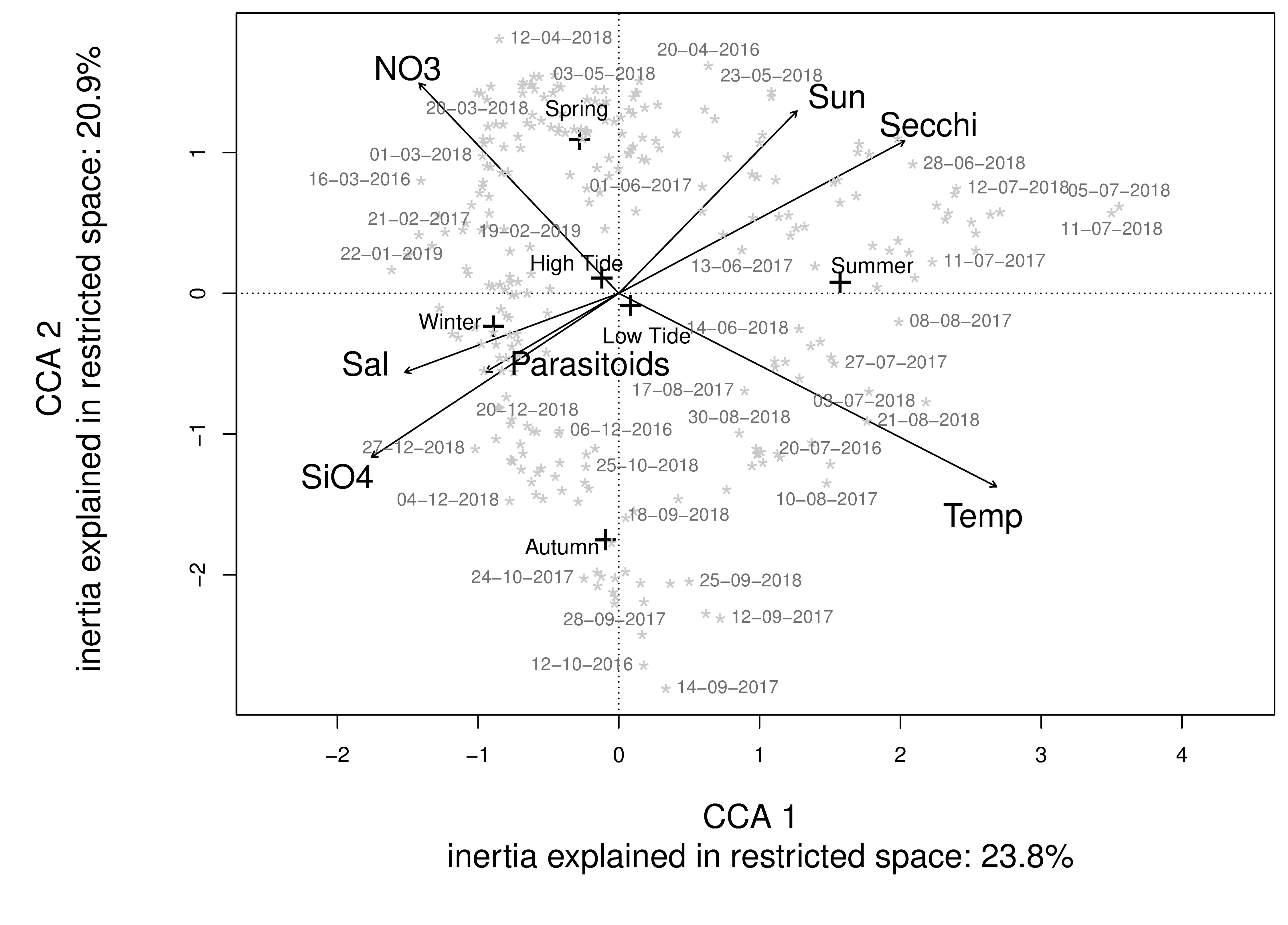

Supplement: S3 Fig — 12.2% of total inertia could be explained by all variables in full space, in restricted space CCA1 explained 23.8% of the variance and CCA2 explained 20.9%. (TIF) [file pone.0244817.s003.tif]

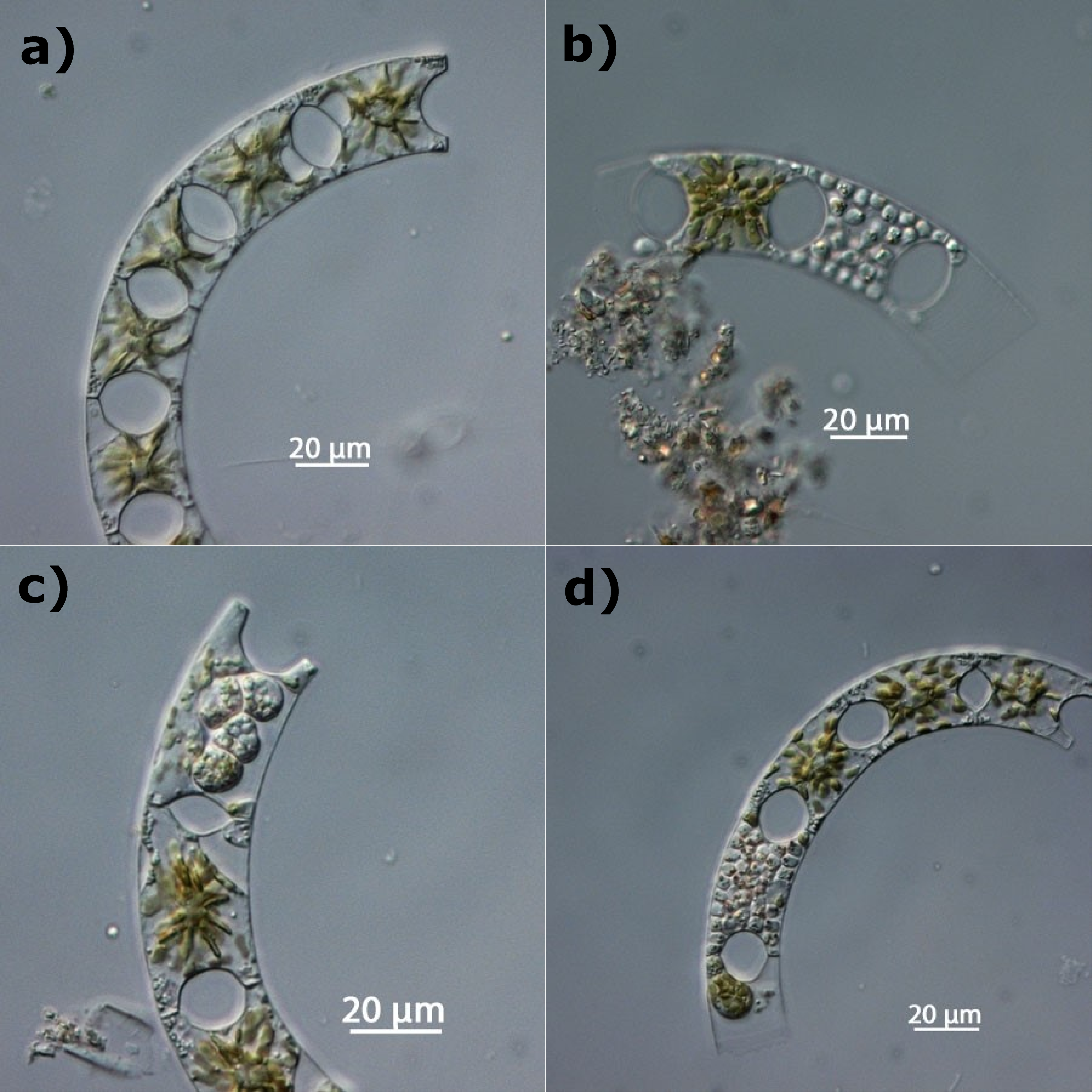

Supplement: S4 Fig — Live cells of the centric diatom Eucampia zodiacus collected at Helgoland Roads, a) without parasitic infection (3rd August 2017), b)-d) with parasitic infection (b) 27th July 2017, c-d) 29th August 2017). Figures retrieved from planktonnet.awi.de. (TIF) [file pone.0244817.s004.tif]

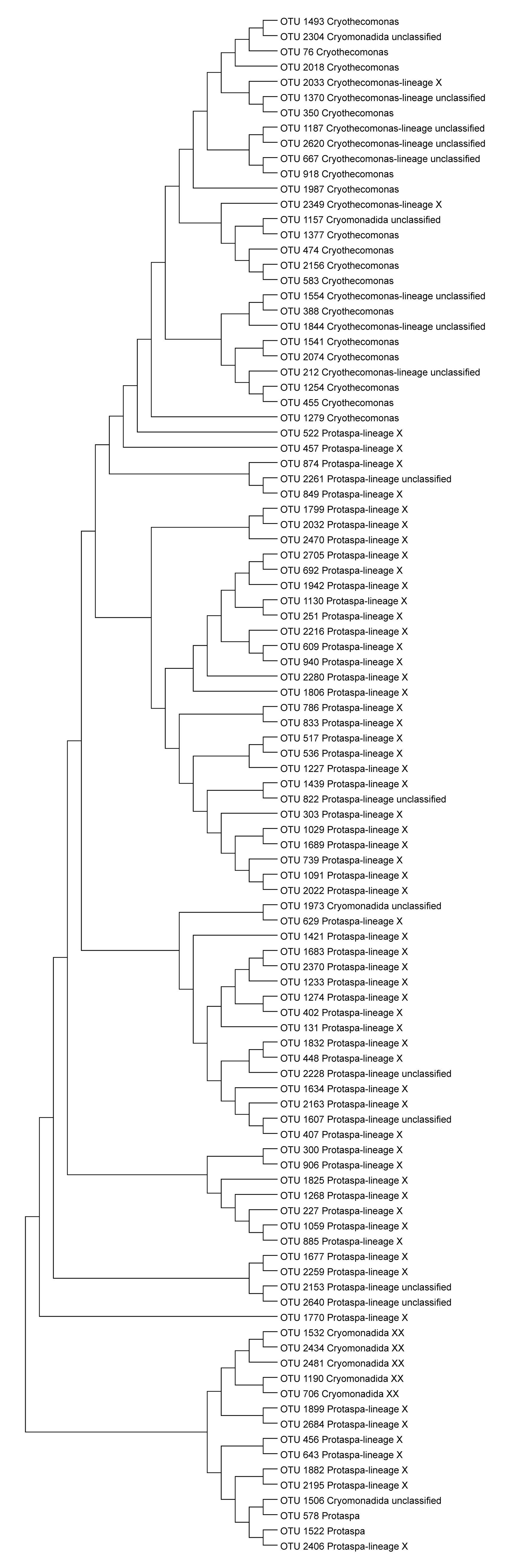

Supplement: S5 Fig — The evolutionary history was inferred by using the Maximum Likelihood method and Tamura-Nei model [101]. The tree with the highest log likelihood (-3807.40) is shown. Initial tree(s) for the heuristic search were obtained automatically by applying Neighbor-Join and BioNJ algorithms to a matrix of pairwise distances estimated using the Tamura-Nei model, and then selecting the topology with superior log likelihood value. This analysis involved 101 nucleotide sequences. There were a total of 397 positions in the final dataset. Evolutionary analyses were conducted in MEGA X [100]. (TIF) [file pone.0244817.s005.tif]
