# Supplementary material for: Host-parasitoid associations in marine planktonic time series: Can metabarcoding help reveal them?
Source: PLoS One. 2021 Jan 7;16(1):e0244817. doi: 10.1371/journal.pone.0244817 (PMC7790432; doi:10.1371/journal.pone.0244817)
Supplement: S1 File — (DOCX) [file pone.0244817.s012.docx]

**S1 File. Additional information on bioinformatic pipeline and analysis**

**Material and Methods**

The chosen pipeline, which is focusing on a relative strict parameter set and a high confidence cut-off of annotation (here named as “default”) aiming for a high reliability, was compared with other settings of the same tools. Additionally, for clustering into operational taxonomic units (OTUs) by the tool swarm (version 2.2.2) [1,2], we compared the results of the default settings with changed up d values (Table 2). Prior to comparison, the threshold of 0.001% of all reads was applied in each dataset.

OTU tables of these additional parameter settings can be found on zenodo.org (DOI: 10.5281/zenodo.4319940).

**Table 1. Different settings and steps of the bioinformatic pipeline. The term “default” refers to the settings as they have been chosen for this study.**

| tool and action | tool version and reference | "relaxed" settings | "default" settings | "strict" settings | "very strict" settings |
| --- | --- | --- | --- | --- | --- |
| trimming with trimmomatic (length sliding window - average quality score within) | version 0.38  [3] | 3-5 | 3-8 | 1-15 | 1-15 |
| merge with vsearch (length of minimum overlap of paired end reads - max number of mismatches allowed) | version 2.3.0  [4] | 25-5 | 50-5 | 50-0 | 50-0 |
| primer removal with cutadapt (max [x*100]% mismatches in at least [y*100]% primer-to-sequence overlap) | version 1.19  [5] | 0.2-0.75 | 0.1-0.9 | 0.1-0.9 | 0-1 |
| eemax-filtration with vsearch (expected error of x allowed in sequence) | version 2.3.0 | 1 | 0.25 | 0.1 | 0.1 |
| length filtration with vsearch (min len x - max len y) | version 2.3.0 | 300-550 | 300-550 | 300-550 | 300-550 |
| classification with mothur (cutoff value of taxonomic level) | version 1.38.1  [6] | 0.6 | 0.8 | 0.9 | 0.9 |

**Table 2. Combinations of different settings for comparison of results. and steps of the bioinformatic pipeline. The term “default” refers to the settings as they have been chosen for this study.**

|  | chosen d value in swarm | parameter settings (relaxed, default, strict, very strict) |
| --- | --- | --- |
| option 1 (this study) | 1 | default |
| option 2 | 2 | default |
| option 3 | 3 | default |
| option 4 | 5 | default |
| option 5 | 10 | default |
| option 6 | 1 | relaxed |
| option 7 | 2 | relaxed |
| option 8 | 3 | relaxed |
| option 9 | 1 | strict |
| option 10 | 2 | strict |
| option 11 | 3 | strict |
| option 12 | 1 | very strict |

**Results**

Higher d values resulted in less OTUs in the datasets, since potential OTUs that are found with lower d values are merged with other close OTUs when distances are too big. Except for the very strict settings, which filters all sequences that do not fit 100% in primer-to-sequence overlap, parasitoid sequences of all 10 phyla were found (Table 3). Identification of the sequences was not possible for the Perkinsea sequence, when (very) strict settings with different d values were used (option 10-12). Additionally, in option 9-12 (marked in red) some samples were removed by the pipeline due to poor quality. For all options, new sequences that were identified as parasitoids were found in the dataset after the 0.001% threshold. In general, the distribution of parasitoids in the different phyla remained similar. For example, parasitoid Dinoflagellata distributed the most OTUs, followed by Cercozoa parasitoids. Additionally, in all options the three most abundant OTUs (including non-parasitoids) belonged to the genera *Paracalanus*, *Temora* and Gyrodinium.

**Table 3. Comparison of results of different pipeline settings. “Old” sequences refer to sequences that are present in the dataset of the 2790 OTUs of the study (option 1).**

|  | number of OTUs (at 0.001% threshold) | number of samples | total Parasitoids (0.001%) | number of "old" / "new" parasitoid sequences found | number of phyla (sequences / identified) | comment |
| --- | --- | --- | --- | --- | --- | --- |
| option 1 (this study) | 2790 | 280 | 461 |  | 10/10 |  |
| option 2 | 2274 | 280 | 414 | 405/9 | 10/10 |  |
| option 3 | 2125 | 280 | 381 | 370/11 | 10/10 |  |
| option 4 | 1921 | 280 | 340 | 324/16 | 10/10 |  |
| option 5 | 1539 | 280 | 274 | 258/16 | 10/10 |  |
| option 6 | 2904 | 280 | 491 | 403/88 | 10/10 |  |
| option 7 | 2313 | 280 | 431 | 353/78 | 10/10 |  |
| option 8 | 2165 | 280 | 405 | 324/81 | 10/10 |  |
| option 9 | 2522 | 267 | 466 | 423/43 | 10/10 |  |
| option 10 | 2208 | 267 | 410 | 375/35 | 10/9 | Perkinsea sequence found but not identified by PR2 |
| option 11 | 2068 | 267 | 379 | 345/34 | 10/9 | Perkinsea sequence found but not identified by PR2 |
| option 12 | 2200 | 266 | 419 | 390/29 | 09/08 | parasitic Metazoa sequences not found/identified, Perkinsea sequence found but not identified by PR2 |

**References**

1. Mahé F, Rognes T, Quince C, de Vargas C, Dunthorn M. Swarm v2: highly-scalable and high-resolution amplicon clustering. PeerJ. 2015;3: e1420. doi:10.7717/peerj.1420

2. Mahé F, Rognes T, Quince C, de Vargas C, Dunthorn M. Swarm: robust and fast clustering method for amplicon-based studies. PeerJ. 2014;2: e593. doi:10.7717/peerj.593

3. Bolger AM, Lohse M, Usadel B. Trimmomatic: A flexible trimmer for Illumina sequence data. Bioinformatics. 2014;30: 2114–2120. doi:10.1093/bioinformatics/btu170

4. Rognes T, Flouri T, Nichols B, Quince C, Mahé F. VSEARCH: a versatile open source tool for metagenomics. PeerJ. 2016;4: e2584. doi:10.7717/peerj.2584

5. Martin M. Cutadapt removes adapter sequences from high-throughput sequencing reads. EMBnet.journal. 2011;17: 10–12. doi:https://doi.org/10.14806/ej.17.1.200

6. Schloss PD, Westcott SL, Ryabin T, Hall JR, Hartmann M, Hollister EB, et al. Introducing mothur: Open-Source, Platform-Independent, Community-Supported Software for Describing and Comparing Microbial Communities. Appl Environ Microbiol. 2009;75: 7537–7541. doi:10.1128/AEM.01541-09
